# Unraveling the Mechanisms of Zirconium Metal–Organic Frameworks‐Based Mixed‐Matrix Membranes Preventing Polysulfide Shuttling

**DOI:** 10.1002/smsc.202300339

**Published:** 2024-05-02

**Authors:** Wenqing Lu, Zhenfeng Pang, Aran Lamaire, Fu Liu, Shan Dai, Moisés L. Pinto, Rezan Demir‐Cakan, Kong Ooi Tan, Veronique Van Speybroeck, Vanessa Pimenta, Christian Serre

**Affiliations:** ^1^ Institut des Matériaux Poreux de Paris ESPCI Paris, Ecole Normale Supérieure, CNRS, PSL University 75005 Paris France; ^2^ Laboratoire des Biomolécules, LBM Département de Chimie École Normale Supérieure, PSL University Sorbonne Université, CNRS 75005 Paris France; ^3^ Center for Molecular Modeling (CMM) Ghent University Technologiepark‐Zwijnaarde 46 9052 Zwijnaarde Belgium; ^4^ Collège de France, Chimie du Solide et de l’Energie—UMR 8260 CNRS 75005 Paris France; ^5^ Réseau sur le Stockage Electrochimique de l’Energie (RS2E)—FR, CNRS 3459 Amiens France; ^6^ CERENA–Centro de Recursos Naturais e Ambiente, Departamento de Engenharia Química, Instituto Superior Técnico Universidade de Lisboa Av. Rovisco Pais, 1 1049‐001 Lisboa Portugal; ^7^ Department of Chemical Engineering Gebze Technical University 41400 Kocaeli Turkey

**Keywords:** interlayers, lithium–sulfur batteries, metal–organic frameworks, mixed‐matrix membranes, modeling, solid‐state nuclear magnetic resonance

## Abstract

Lithium–sulfur batteries are considered as promising candidates for next‐generation energy storage devices for grid applications due to their high theoretical energy density. However, the inevitable shuttle effect of lithium polysulfides and/or dendrite growth of Li metal anodes hinder their commercial viability. Herein, the microporous Zr fumarate metal–organic framework (MOF)‐801(Zr) is considered to produce thin (≈15.6 μm, ≈1 mg cm^2^) mixed‐matrix membranes (MMM) as a novel interlayer for Li–S batteries. It is found that the MOF‐801(Zr)/C/PVDF‐HFP composite interlayer facilitates Li^+^ ions diffusion, and anchors polysulfides while promoting their redox conversion effectively. It is demonstrated that MOF‐801 effectively trapped polysulfides at the cathode side, and confirmed for the first time the nature of the interaction between the adsorbed polysulfides and the host framework, through a combination of solid‐state nuclear magnetic resonance and molecular dynamics simulations. The incorporation of MOF‐801(Zr)/C/PVDF‐HFP MMM interlayer results in a notable enhancement in the initial capacity of Li–S batteries up to 1110 mA h g^−1^. Moreover, even after 50 cycles, a specific capacity of 880 mA h g^−1^ is delivered.

## Graphical abstract

Microporous metal–organic frameworks‐801(Zr) is a model to decipher the nature of the interaction between the adsorbed polysulfides and the host framework, through a combination of solid‐state nuclear magnetic resonance and molecular dynamics simulations and its shaping in mixed‐matrix membranes as a novel interlayer material for lithium‐sulfur batteries.

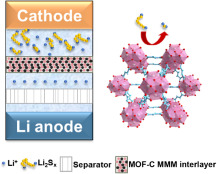

## 1 Introduction

Lithium–sulfur batteries (Li–S) are considered as one of the most promising next‐generation energy storage systems for grid applications due to their high energy density and cost‐effectiveness.^[^ ^1^, ^2^, ^3^, ^4^, ^5^, ^6^ ^]^ However, a few drawbacks still limit their commercial application, such as their capacity fading and/or short cycle life due to the shuttle effect of soluble lithium polysulfides.^[^ ^7^, ^8^, ^9^, ^10^ ^]^ To mitigate this problem, the modification of conventional separators (e.g., glass fiber or Celgard) by including microporous materials has been proposed. Although this strategy has been extensively studied so far, modified separators are usually intrinsically limited, since the cohesion between the rigid functional material and the flexible separator is poor.^[^ ^11^, ^12^, ^13^ ^]^ Another strategy that in principle might overcome this limitation consists of the use of an interlayer, usually a self‐standing film, placed between the sulfur cathode and the anode. At present, there are few reports about interlayers, and the existing interlayers are often composed of porous or dense conductive carbons.^[^ ^14^, ^15^ ^]^ Nonetheless, due to the weak adsorption between nonpolar carbon‐based materials and polar lithium polysulfides (LPS), the shuttle effect cannot be effectively suppressed.

Metal–organic frameworks (MOFs) are one of the latest classes of ordered micro‐ or mesoporous hybrid materials with highly tunable structural and/or chemical characteristics suitable for a wide range of applications from gas sorption, separation, catalysis, sensing to biomedicine, among others.^[^ ^16^, ^17^, ^18^, ^19^ ^]^ This makes them highly promising candidates to limit polysulfides migration due to their unique structural and chemical properties, such as high porosity, uniform and tunable pore size, Lewis/Bronsted acidity, and structural defects, which shall significantly impact their molecular sieving ability.^[^ ^20^, ^21^, ^22^, ^23^ ^]^ MOFs are expected here to play different roles: 1) as an ion sieving medium to allow selectively lithium ions to pass through while 2) preventing polysulfide ions from shuttling. Nonetheless, there is no consensus on what are the ideal parameters, e.g., particle size^[^ ^24^, ^25^ ^]^ or structural features,^[^ ^12^, ^26^, ^27^ ^]^ to fulfill this role while clear‐cut mechanistic studies are still lacking. Zirconium‐based MOFs are a subset class of robust MOFs with a rare combination of chemical/thermal stability and highly versatile chemical/structural tunability; among a series of benchmark structures, the prototypical Zr terephthalate UiO‐66 has been studied for Li–S batteries due to its micropores of 8–11 Å, the presence of structural defects which yield polar sites, and the possibility of incorporate polar functional groups in the linker. The combination of these features was shown to enhance polysulfides confinement.^[^ ^11^, ^28^, ^29^ ^]^ However, even with the structural defects and the introduction of functional groups, the length of the linker (terephthalate) limits the tuning of smaller pore sizes, requiring the development of stable materials with smaller pore size and higher density of active sites.

The benchmark Zr fumarate MOF‐801(Zr) has been considered here as an alternative candidate owing to its narrow pores and its “polar” character, due to the high content of structural defects and the presence of numerous terminal –OH groups in the Zr oxo‐cluster. Its three‐dimensional cubic rigid structure, resulting from the assembly of Zr_6_O_4_(OH)_4_(CO_2_)_12_ secondary building units and fumarate linkers, exhibit indeed smaller micropores (about 5–7 Å) than UiO‐66 while possessing a large number of ligand defects particularly when a green low‐pressure synthesis protocol is followed.^[^ ^30^, ^31^, ^32^ ^]^ This results in a large surface area of microporous material with a highly polar character, due to the presence of highly reactive acidic Zr‐OH groups from the Zr_6_ oxo‐clusters. Structural defects are also expected to be beneficial to further limit the polysulfides migration.^[^ ^32^, ^33^ ^]^ Moreover, Zr‐ and Ce‐based MOFs have already been reported as catalytically active for the conversion of long‐chains polysulfides in short‐chains species, which enhances the electrochemical performance of the Li–S cells.^[^ ^34^, ^35^ ^]^ Nonetheless, the use of the sole pristine MOFs as an interlayer presents several limitations: on one hand, the nonconductive nature of most MOFs limits the electron percolation and reactivation of the sieved polysulfides, associated with a poor Li‐ion migration kinetics; on the other hand, pure MOF membranes present poor mechanical properties and often numerous defects. To circumvent the mechanical properties limitation, a mixed‐matrix membrane (MMM) approach is a sound approach. These composite membranes consist of an organic polymer matrix phase and an inorganic or hybrid solid filler phase, combining the required characteristics of these different materials and yielding materials with tailored permeability and selectivity.^[^ ^36^ ^]^ However, developing flexible interlayers that can effectively mitigate polysulfides and facilitate Li^+^ transport to achieve high battery performance is still an ongoing challenge.

Recently a MOF‐801(Zr)‐carbon cloth composite has been proposed as interlayer for Li–S devices.^[^ ^37^ ^]^ The MOF‐801 particles were directly grown into the carbon cloth support through an in situ solution crystallization method. Nonetheless, although one can observe the mitigation of the polysulfides shuttling to a certain extent, the mechanical stability of the MOF film at the surface of the carbon fibers was not discussed by the authors. Moreover, the host–guest interaction between the LPS was poorly addressed. In our case, we have designed a flexible free‐standing MOF‐based interlayer with high mechanical and chemical stability through a MMMs approach. This strategy allows to combine the chemical features of the three components of the composite: a) MOF‐801, a robust microporous Zr‐based MOF with a large pore volume and abundantly exposed Zr‐OH active sites expected to enhance the polysulfides anchoring while facilitating Li^+^ ions transport; b) Ketjen black (KB) carbon, a highly conductive carbon to enhance the electrical conductivity and improve the reactivation of sulfur species hosted in the MOF's pores, while participating to the adsorption process due to its high surface area (around 1200 m^2^ g^−1^); c) Poly(vinylidene fluoride‐co‐hexafluoropropylene) (PVDF‐HFP), a polymer commonly used in battery field, known for its great mechanical properties and for allowing high filler loadings. The composite membranes were optimized in terms of porosity and mechanical properties and in‐depth characterized through a combination of structural and morphological techniques, to understand the synergy between all components of the membrane, as well as solid‐state nuclear magnetic resonance (NMR) and molecular dynamics (MD) to shed light on the polysulfides adsorption mechanism. Finally, the electrochemical behavior of Li–S cells containing MOF‐801(Zr)/C/PVDF interlayer was studied and a significant improvement of the specific capacity (880 mA h g^−1^ after 50 cycles) was found when compared to the cells containing only the routine glass fiber separator.

## 2 Results and Discussion

### 2.1 Synthesis and Polysulfides Adsorption Test of MOF‐801(Zr)

**Figure 1:**
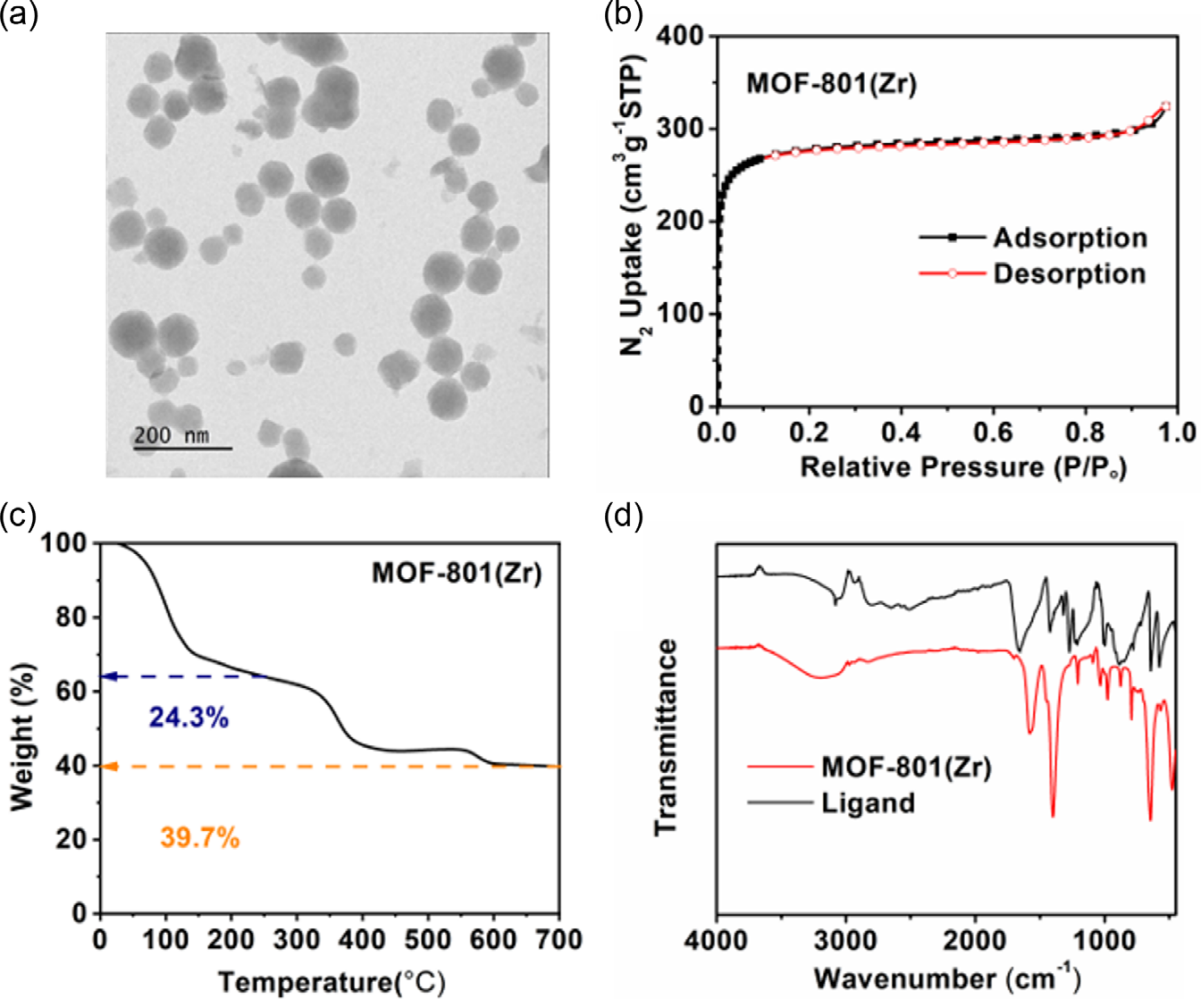
a) TEM images of MOF‐801(Zr) nanoparticles; b) N_2_ adsorption–desorption isotherms at 77 K (*P*_0_ = 1 atm) of MOF‐801(Zr); c) TGA of MOF‐801(Zr) under oxygen atmosphere (heating rate of 3 °C min^−1^); and d) Fourier‐transform infrared spectroscopy (FT‐IR) spectra of MOF‐801(Zr) and fumaric acid (ligand).

MOF‐801(Zr) nanoparticles were prepared through a room‐temperature synthesis method already reported by us.^[^ ^32^ ^]^ The powder X‐ray diffraction (PXRD) pattern (Figure S1a, Supporting Information) of the obtained MOF‐801(Zr) was found to be in good agreement with the simulated one, confirming the purity and crystallinity of the obtained compound. The transmission electron microscopy (TEM) (**Figure** **1**a) and scanning electron microscopy (SEM) (Figure S1b, Supporting Information) images showed that MOF‐801(Zr) nanoparticles exhibit a spherical crystal morphology with a particle size dispersion comprised between 40 and 80 nm, in agreement with the literature.

To investigate the microstructure of MOF‐801(Zr), N_2_ adsorption–desorption analysis (Figure 1b) was performed at 77 K, resulting in a high specific surface area of 1020(±20) m^2^ g^−1^, calculated through the Brunauer–Emmett–Teller (BET) method. In addition, the pore size distribution (PSD) (Figure S1c, Supporting Information) calculated by the nonlocal density functional theory (NLDFT) (‐N_2_ method, revealed the microporosity (5–13 Å) of the synthesized MOF‐801. The expanded PSD correlated further confirming the defective nature of MOF‐801(Zr). Hence, these results have demonstrated the suitability of MOF‐801(Zr) as an ideal molecular sieve for blocking large‐sized Li_2_S_8_ (≈2 nm length)^[^ ^14^ ^]^ while letting the lithium ions (ionic diameters: 0.12 nm) pass.^[^ ^31^ ^]^ Based on the residual content of ZrO_2_ obtained from thermogravimetric analysis (TGA), the ligand connectivity of the Zr_6_ oxocluster was calculated to be 7.94, which contrasted with the theoretical connectivity of 12 for the defect‐free structure. This calculation supported the presence of a significant number of missing ligands within the solid. Additionally, following recent systematic studies on MOF defect quantification,^[^ ^32^, ^38^ ^]^ we decomposed the MOF in an alkaline medium and conducted ^1^H NMR analysis. As shown in Figure S1d (Supporting Information), a substantial amount of formate was observed, with a formate: fumarate molar ratio of 1:4.73, indicating a significant replacement of the fumarate linker by formate. Combining these results with the N_2_ adsorption data (1200 m^2^ g^−1^ in this case compared to 650 m^2^ g^−1^ for defect‐free MOF‐801), we could conclude that the synthesized MOF‐801 possessed a large number of defects. The FT‐IR spectra showed a large number of vibrations of –OH groups and strong vibrations associated to COO^−^ groups coordinated to Zr^IV^, while almost no trace of uncoordinated fumaric acid (≈1644 cm^−1^) was observed, which further indicated that the as‐prepared pure MOF‐801(Zr) possess a large number of terminal or bridging Zr‐OH/‐H_2_O polar groups suitable to interact strongly with polysulfides of various lengths.

**Figure 2:**
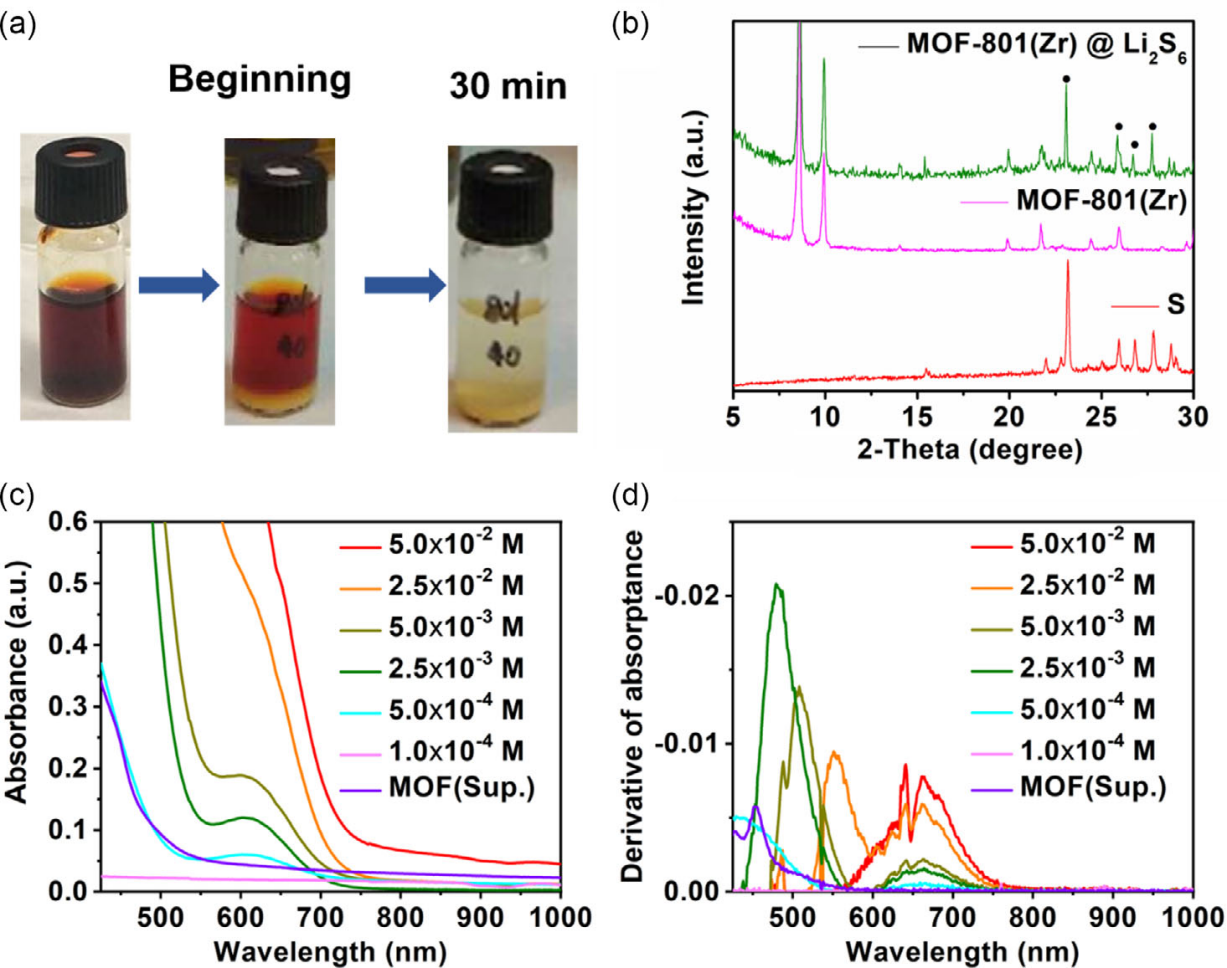
a) Polysulfides adsorption test; b) PXRD pattern of powder samples before and after polysulfide adsorption test (*λ*_Cu_ ≈ 1.5406 Å); c) UV−vis absorption spectra of Li_2_S_6_ at different concentrations solutions and 0.05 M Li_2_S_6_ solution after adding MOF‐801 for 24 h which named MOF(Sup.); and d) first derivatives of the UV−vis spectra.

To investigate the capability of MOF‐801(Zr) to efficiently adsorb polysulfides, 50 mg of MOF‐801(Zr) was added into 1 mL of 0.05 M Li_2_S_6_ solution. Within 30 min, the solution became colorless. The MOF powder, initially white, turned yellowish in polysulfide‐containing solution, suggesting the strong interaction of the Zr‐oxoclusters with polysulfides (**Figure** **2**a). After self‐standing for one week, the structural integrity of MOF‐801 exposure to polysulfides was confirmed by PXRD (Figure 2b). Characteristic reflections of elemental sulfur could be observed, nonetheless, this was certainly ascribed to the crystallization of sulfur during the sample drying, as for the wet sample no sulfur peaks were observed (Figure S1e, Supporting Information). Different concentrations of polysulfides were in parallel tested to determine the detection limit of the dissolved polysulfides by UV−vis spectroscopy (Figure 2c).

To better discriminate the solutions with different polysulfide concentrations, the first derivatives of the UV−vis spectra were compared.^[^ ^39^, ^40^ ^]^ For each concentration, typical peaks in the UV−vis spectra derivative could be observed, with a peak shift to the UV region as the concentration decreases. As shown in Figure 2d, the concentration of 0.05 M Li_2_S_6_ solution decreased to 5 × 10^−4 ^M after adding MOF‐801 for 24 h, which further quantitatively demonstrates the ability of MOF‐801(Zr) to adsorb polysulfides. This could be ascribed to the microporous structure nature of MOF‐801 and the presence of abundant polar sites within the pores.^[^ ^7^, ^41^ ^]^ The adsorption properties of MOF‐801 after incorporation in a PVDF‐HFP matrix have also been tested (Figure S2, Supporting Information). When compared with the base PVDF‐HFP membrane, the MOF‐based membrane effectively limits the diffusion of the polysulfide species.

### 2.2 Solid‐State NMR

Although numerous studies about polysulfides selective adsorption with MOF‐based modified separators or interlayers can be found in the literature, very few reported the study on the host‐guest interactions. To shed light on this phenomenon, magic‐angle spinning (MAS) solid‐state NMR experiments were performed on ^1^H, ^7^Li, and ^13^C.

**Figure 3:**
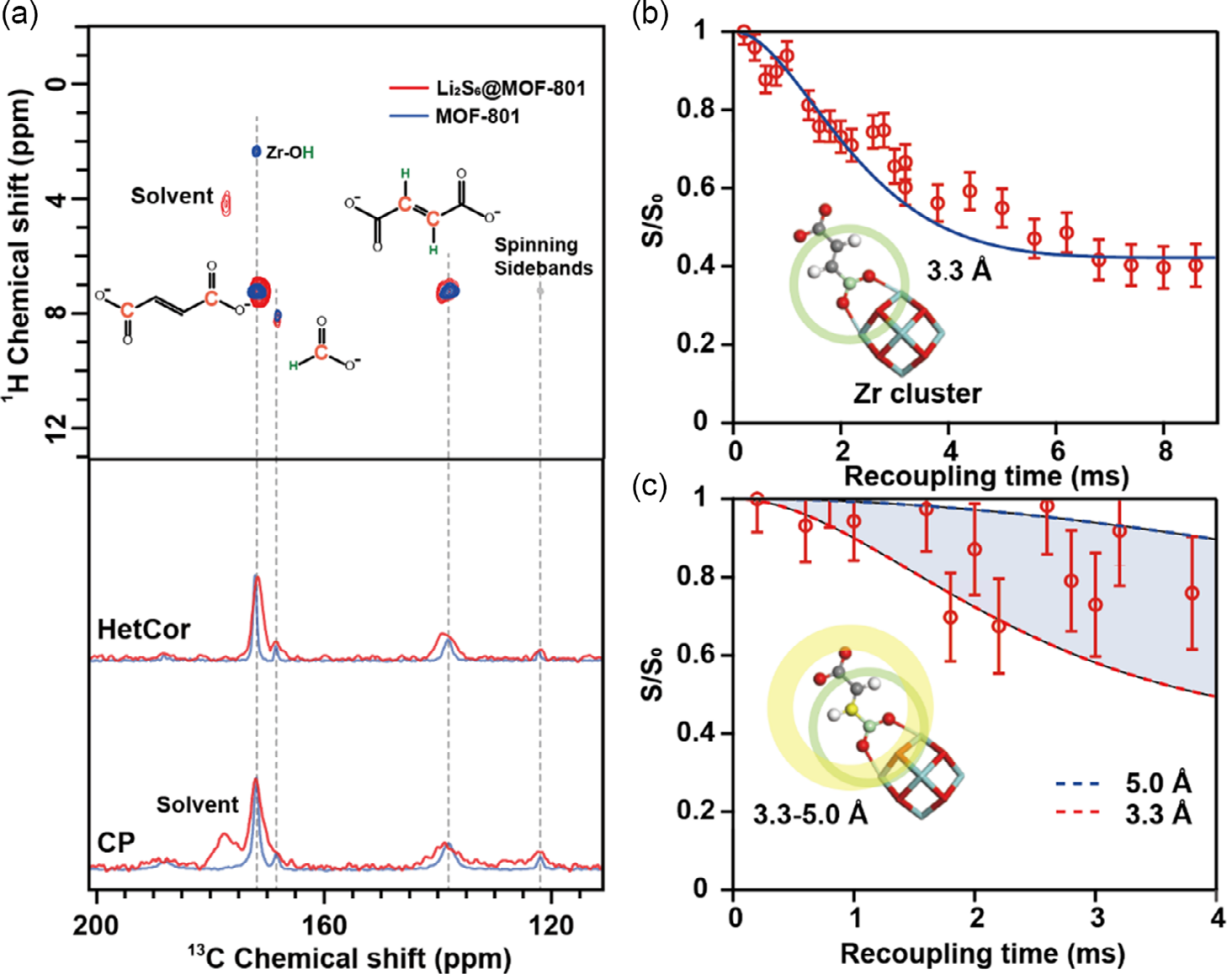
Solid‐state NMR results. a) ^1^H‐^13^C HETCOR spectra (top) of pristine MOF‐801(Zr) (blue) and Li_2_S_6_‐loaded MOF‐801 (red), and projection of 2D HETCOR spectra to the ^13^C dimension (middle) and ^1^H‐^13^C CP spectra (bottom). ^7^Li‐^13^C REDOR dephasing curves due to the coupling of lithium and carboxyl carbon (≈3.3 Å); b) or the coupling of lithium and double bond carbon (≈3.3–5 Å); c) of fumarate in Li_2_S_6_‐loaded MOF‐801. The possible lithium positions are highlighted by the faint yellow and green shells and the yellow atom refers to the carboxylate carbon atom.

As shown in **Figure** **3**a, the peak widths of fumarate (13C: ≈172 ppm for carbonate, ≈138 ppm for double bond; 1H: ≈7.0 ppm for double bond) and formate (13C: ≈168 ppm; 1H: ≈8.0 ppm) became broader upon Li_2_S_6_ absorption. Note that the traces of formate groups were still found (see Figure S3 for assignment, Supporting Information)^[^ ^42^ ^]^ originating from the formic acid used during the MOF synthesis and not completely removed during the washing process. In the Li_2_S_6_‐loaded MOF‐801(Zr), a new ^13^C site of around 175 ppm emerged and it can be attributed to the thermal decomposition of dioxolane solvent during the preparation of the polysulfides solution since only a correlation between the new ^13^C site and the proton from solvent was observed. The observed NMR line‐broadening effect implies that the MOF‐801(Zr) has experienced a change in the chemical environment due to the loading of Li_2_S_6_ within the pores. The interaction between Li and the MOF‐801 framework was also confirmed by the 1D ^7^Li spectrum and the 2D ^7^Li‐^1^H correlation experiment (Figure S4, Supporting Information), where the adsorbed ^7^Li and the cross peaks between lithium and fumarate are observed. To locate lithium in MOF‐801(Zr), ^7^Li‐^13^C REDOR experiments were performed to determine the distances between lithium and various carbon sites. As shown in Figure 3b, the nearest lithium is 3.3 ± 0.5 Å to the carbon of carboxylate (–^13^COO) in fumarate. Following that, it can be estimated that the distance between the ^7^Li and the double bond ^13^C signal (^13^C=^13^C) is between 3.3 and 5.0 Å (Figure 3c). The measurement is less precise in the latter case due to the low sensitivity resulted from the line‐broadening effect. More accurate distances are expected to be obtained by using ^13^C‐labelled fumaric acid for the synthesis of the MOF.

Hence, the results suggest that the lithium atoms are located within the overlapped zone bounded by the two shells in Figure 3b,c. This indicates that lithium is rather closer to Zr_6_ oxoclusters, and, hence, closely interacting with the carboxylate group. Moreover, the ^13^C centerband‐only detection of exchange (CODEX) result (Figure S5, Supporting Information) suggests that there are two lithium staying close to each other, i.e., there is only a single (not a cluster) lithium polysulfide chain in the vicinity. Nonetheless, due to the lack of reliable measurements on reference samples, the exact distance between the two lithium atoms could not be determined, which could have helped to identify the shape of the chain.

### 2.3 MD Simulations

Additional insight into the host–guest interactions between MOF‐801 and Li_2_S_6_ could be obtained from MD simulations. The MOF‐801 model structure used in the simulations has Zr_6_ oxoclusters with an eightfold linker connectivity, in agreement with the experimental measurements where a ligand‐to‐metal ratio close to 8 was deduced. The most straightforward approach to simulate an eightfold linker connectivity consists of removing one Zr_6_ oxocluster along with the 12 attached linkers from the pristine MOF‐801 unit cell. This merges the tetrahedral pores of MOF‐801 into a larger pore with a diameter of about 13.5 Å (Figure S6, Supporting Information). The remaining metal nodes in the unit cell are terminated with formate.

**Figure 4:**
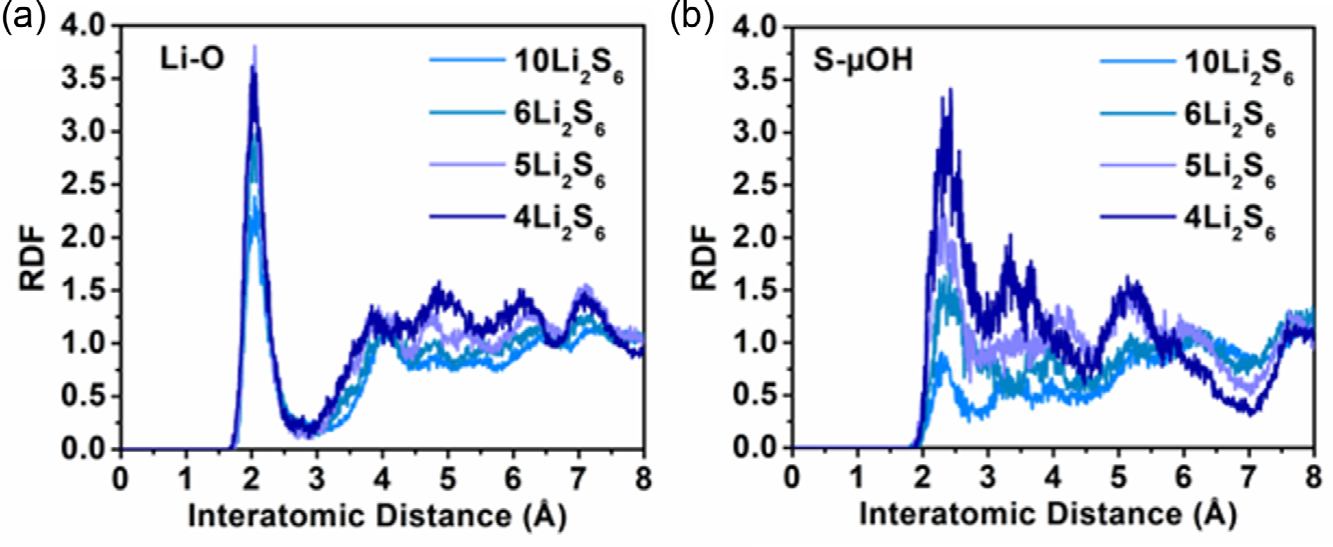
RDFs for a) Li—O and b) S—H_μOH_ atom pairs obtained from first‐principles MD simulations of MOF‐801 with different Li_2_S_6_ loadings.

To probe both the host–guest and guest–guest interactions for MOF‐801 and Li_2_S_6_, different Li_2_S_6_ loadings were considered (ranging from 4 to 10 Li_2_S_6_ molecules per unit cell). The dominant interactions could be identified by calculating radial distribution functions (RDFs) for different atom pairs. The lithium atoms were shown to interact primarily with the oxygen atoms of the fumarate linkers and the formate groups grafted to the Zr6 oxocluster, resulting in an RDF peak at about 2 Å (**Figure** **4**a), which confirmed the host–guest interactions also observed in the NMR experiments. For sulfur, the interaction with the μ‐OH groups on the metal nodes was most pronounced, resulting in an RDF peak at about 2.3 Å (Figure 4b). When different Li_2_S_6_ molecules are in close proximity during the MD simulation, they also showed a strong tendency to interact with one another to form a larger polysulfide cluster.

Also for a second MOF‐801 model with a ninefold linker connectivity, which is obtained by only removing linkers from the pristine structure, similar conclusions could be drawn. As the metal nodes in this second model are terminated with OH‐groups and a water molecule instead of with formate, the Li‐O RDF peak at about 2 Å now contains contributions from the oxygen atoms of both the fumarate linkers and the –OH/H_2_O terminations of the metal nodes (Figure S7, Supporting Information). Similarly, the sulfur atoms can now interact with the μ‐OH groups on the metal nodes as well as the additional –OH/H2O terminations (Figure S7, Supporting Information).

This new approach, based on a combination of experimental data from solid‐state NMR and MD simulation, allowed to unravel for the first time the preferential interaction of the polysulfides with the host MOF structure. The lithium atoms preferentially interact with the carboxylate groups, as sustained by the ^7^Li–^13^C coupling observed by solid‐state NMR and by the Li–O peak in the RDF. The S‐μ‐OH interaction from RDF also sustains a close interaction of the polysulfides with the Zr6 oxocluster, through the terminal acidic Zr‐OH groups.

### 2.4 Preparation of MOFs‐Based MMM Interlayers

To investigate the efficiency of MOF‐801(Zr) in Li–S devices, a functional free‐standing interlayer material was prepared through a MMM approach. MOF‐801(Zr)‐based composite membranes with good flexibility and enhanced electrical conductivity were designed, to overcome the mechanical brittleness and insulator character of the pristine MOF.^[^ ^43^ ^]^ To reach that goal, a three‐component composite was investigated, combining MOF‐801 nanoparticles for the sieving and shuttle effect mitigation, KB particles for the conductivity, and a polymer for the mechanical processability (Table S2, Supporting Information).

MOF‐801‐based MMMs with flat surfaces were first prepared through a blade casting method and recovered as mechanically stable self‐standing films (Figure S8a, Supporting Information). Flexible polymers are known to easily wrap MOF nanoparticles, provided that their respective charge surface displays opposite values, which enhances the attraction between both moieties. Such parameter is crucial for obtaining defect‐free membranes since the particles should be homogeneously distributed within the membrane without agglomerate. As demonstrated for PVDF or poly (ethylene oxyde) (PEO) and UiO‐66, the end groups of polymers can penetrate to some extent into the pores of the MOF, depending on the chemical affinity between the polymer chains and the MOF.^[^ ^44^ ^]^ The main possible drawback is pore blocking, leading in some cases to a strong decrease of accessible porosity.^[^ ^45^ ^]^ To confirm the interactions between PVDF‐HFP and MOF‐801(Zr) nanoparticles, N_2_ porosimetry analysis of MOF‐801(Zr) MMMs was performed. For 30 wt% MOF‐801(Zr) 70 wt% PVDF‐HFP MMMs, if the MOF's pore is not blocked at all, the BET‐specific surface area should be around 300 m^2^ g^−1^, nonetheless, the experimental value is only 167(±1) m^2^ g^−1^ (Figure S8b, Supporting Information), indicating that some polymer chains enter the pores of the MOF and partially block access to the N_2_ molecules at 77 K, in agreement with what was already observed for UiO‐66/PVDF system.^[^ ^44^ ^]^ Note that the MOF‐801(Zr) after the introduction in the membrane retains its crystalline structure (Figure S9, Supporting Information). As the polymer chains partially enter into the pores, further reducing the pore size, this is a priori a favorable parameter to physically block polysulfides, while still enabling a fast Li^+^ (ionic diameters: 0.12 nm) conduction.^[^ ^46^ ^]^

**Figure 5:**
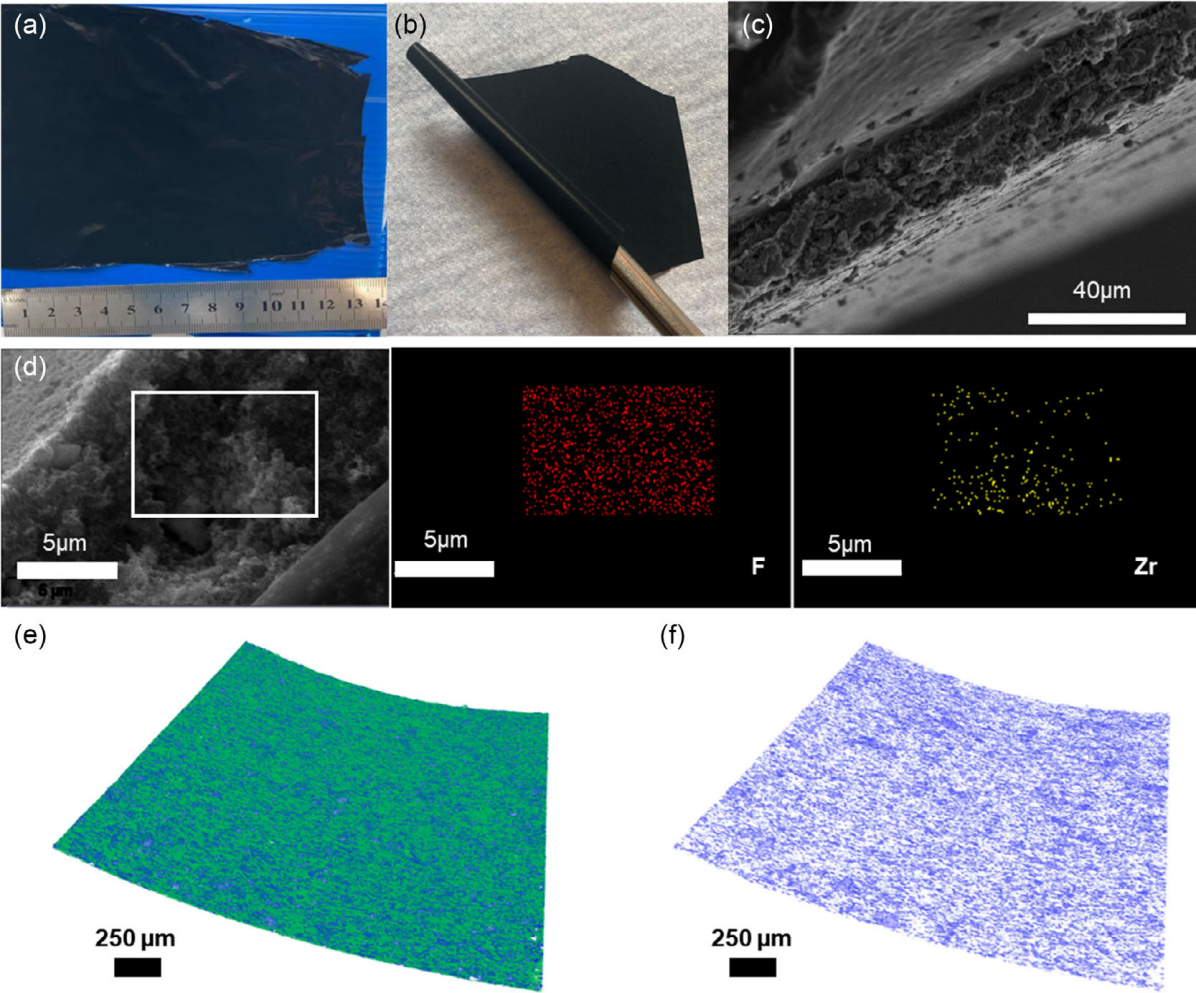
a,b) Photographs of the MOF‐801(Zr)/C/PVDF‐HFP MMM (30 wt% KB); c) cross‐sectional SEM image of the MOF‐801(Zr)/C/PVDF‐HFP MMM; d) cross‐sectional SEM image of the MOF‐801(Zr)/C/PVDF MMM and the corresponding elemental mapping images of F and Zr. e,f) 3D X‐ray microscopy images of the composite membrane MOF‐801(Zr)/C/PVDF MMM (30 wt% KB) with the mapping of the MOF‐801(Zr) particles spatial distribution within the composite membrane in blue.

To prepare conducting membranes, KB was also added to the composite. To determine the optimal ratio of MOF and KB in the membrane, samples containing 10, 20, and 30 wt% KB were prepared (**Figure** **5** and S10–S12, Supporting Information), and their electrochemical behavior was characterized by galvanostatic cycling tests. Figure S12 (Supporting Information) shows that the cell with the interlayer with 30 wt% KB and 30% MOF‐801(Zr)/PVDF‐HFP MMM exhibits the highest specific capacity and stability. Thus, the optimized MMM of 30 wt% KB was adopted in the following studies, named MOF‐801(Zr)/C/PVDF‐HFP MMM, hereafter. Noteworthy, the flexible MOF‐801(Zr)/C/PVDF‐HFP MMM can be curled at will and has excellent mechanical properties (Figure 5b). The SEM images showed that the membrane is flat with a thickness of 15.6 μm (Figure 5c). Inside the membrane (Figure S13, Supporting Information), MOF nanocrystals and KB nanoparticles look tightly packed and the voids between the particles are filled up with PVDF‐HFP polymer, forming a quite dense composite. The SEM elemental mapping (Figure 5d) showed uniform distributions of F and Zr in MMM, demonstrating that PVDF‐HFP and MOF‐801(Zr) form a fairly even distribution.

Visualization of the internal structure of MMMs is of great interest in materials science to get a better understanding of the membrane's mechanical stability and its properties.^[^ ^47^, ^48^, ^49^ ^]^ In this context, 3D X‐ray microscopy is a powerful tool that allows for high‐resolution imaging of the internal structure of MMMs. As shown in Figure 5e, 3D X‐ray microscopy images revealed a uniform distribution of MOF‐801(Zr) and carbon particles within the polymer matrix. Despite the presence of a limited number of particle aggregates (Figure 5f), the overall distribution looks on the whole homogeneous, indicating a well‐dispersed particle configuration. Furthermore, the quantitative analysis of the membrane revealed a MOF‐801(Zr) content of approximately 28 vol%, in good agreement with the initial content of 30 wt% added during the membrane preparation, considering that the MOF particles are denser than the KB and PVDF‐HFP polymer. This confirms the robustness of the fabrication process used in this study, and that MMM with a homogeneous particle distribution could easily be prepared.

**Figure 6:**
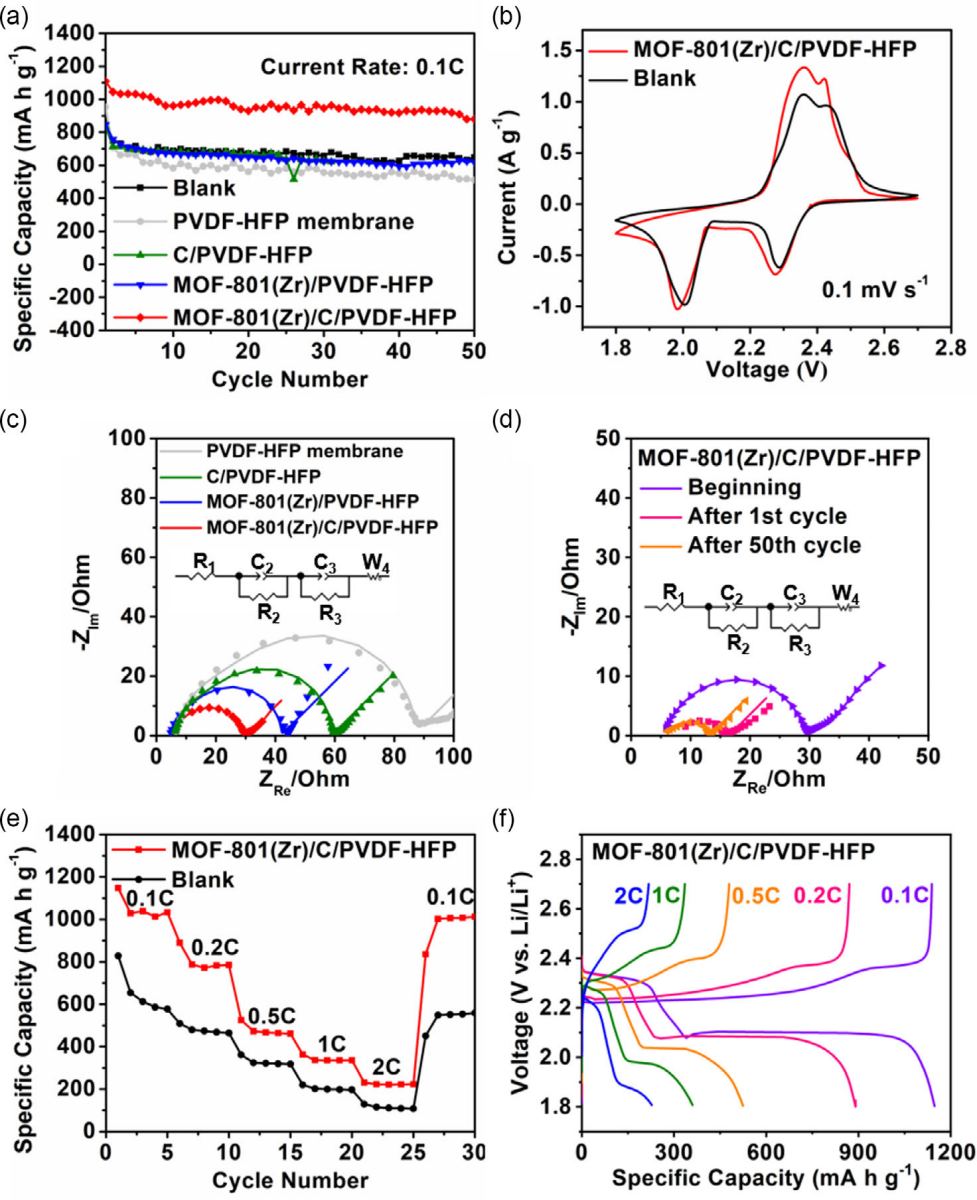
a) Galvanostatic cycling performance at 0.1 C; b) the CV profile of Li–S batteries with and without the MOF‐801(Zr)/C/PVDF‐HFP MMM interlayer at 0.1 mV s^−1^; c) EIS profiles (dots are raw data, lines are fitted data). d) EIS profiles; e) galvanostatic cycling performance of batteries with the MOF‐801(Zr)/C/PVDF‐HFP MMM interlayer and without interlayer (blank) at different current densities; and f) galvanostatic charge–discharge curves at different current densities.

To evaluate the effectiveness of the composite interlayer in the “shuttle effect” mitigation, the electrochemical behavior of Li–S cells was finally evaluated. The self‐sustained composite membrane was placed between the cathode and the routine glass fiber separator and cycled against lithium metal. **Figure** **6**a shows the galvanostatic charge–discharge cyclic behavior of the batteries at 0.1 C.

The batteries with MOF‐801(Zr)/C/PVDF‐HFP MMM interlayer exhibited an initial capacity of 1110 mA h g^−1^, while a high discharge capacity of 880 mA h g^−1^ was still retained after 50 cycles. The reproducibility of these results over 3 cells is shown in Figure S14 (Supporting Information). For comparison, the blank, the PVDF‐HFP, and the MOF‐801(Zr)/PVDF‐HFP MMM delivered 647, 511, and 620 mA h g^−1^, respectively, over 50 cycles. The higher capacity retention indicates that the MOF‐801(Zr)/C/PVDF‐HFP MMM interlayer was able to effectively improve the cycling performance of Li–S batteries. The FT‐IR spectrum of the MOF‐801(Zr)/C/PVDF‐HFP MMM interlayer after 50 cycles did not show any ligand's peak, confirming the stability of MOF‐801(Zr) particles after cycling (Figure S15, Supporting Information). Furthermore, although it is hard to establish a direct comparison between this work and the ones found in the literature for UiO‐66(Zr) based membranes^[^ ^11^, ^28^ ^]^ one can note that for the MOF‐801/C/PVDF‐HFP membrane after 50 cycles the capacity retention (880 mA h g^−1^) is higher than the one observed for a pristine UiO‐66 layer deposited in a polypropylene separator (720 mA h g^−1^ at 0.5 C after 500 cycles) and slightly lower than a UiO‐66/super P based functional separator (964 mA h g^−1^ at 0.5 C after 200 cycles). These discrepancies can be attributed to several factors: 1) not the same membrane architecture ‐ no interlayers but functional separators; 2) different cycling rates (0.5 C vs 0.1 C in this work); 3) the content and the type of conductive carbon, and 4) the different structural features of these two frameworks (not the same linker, defects ratio).

In Figure 6b, two oxidation peaks and two reduction peaks could be observed in the cyclic voltammetry (CV) curves of Li–S batteries with MOF‐801(Zr)/C/PVDF‐HFP MMM interlayer. The reduction peaks at about 2.28 and 1.98 V correspond to the reduction of S_8_ to Li_2_S_*n*_ (4 ≤ *n* ≤ 8) and Li_2_S_*n*_ (4 ≤ *n* ≤ 8) to Li_2_S/Li_2_S_2_, respectively.^[^ ^50^ ^]^ In addition, the peaks of the cell with the MOF‐801(Zr)/C/PVDF‐HFP MMM interlayer were shown to be sharper, and the peak currents were larger than the batteries without the interlayer, demonstrating that the MOF‐801(Zr)/C/PVDF‐HFP MMM interlayer improves the electrochemical kinetics of Li_2_S_*n*_ conversion. The kinetic improvement by this MMM was also confirmed by the electrochemical impedance spectroscopy (EIS) results. The EIS Nyquist plots of batteries with equivalent circuits are shown in Figure 6c, R1 reflects the contact resistance between the electrode and the electrolyte, R_2_ reflects the resistance of charges transfer at the interface of the cathode, and R_3_ reflects interfacial layer resistance at the surface of the anode. The best charge‐transfer capability of this optimized interlayer was evidenced by the lowest R_2_ value (6.827 Ω) than that of the other membranes: MOF‐801(Zr)/PVDF‐HFP MMM (6.863 Ω), C/PVDF‐HFP MMM (11.01 Ω), and PVDF‐HFP membrane (18.07 Ω) (Table S3, Supporting Information). Such a low value can tentatively be explained by the abundant exposed O active sites and Zr‐OH sites on Zr6 oxo‐cluster limiting polysulfides and promoting the sulfur redox reaction kinetics, as sustained by solid‐state NMR and MD calculation results. Furthermore, as shown in Figure 6d and Table S4 (Supporting Information), the impedance value of the MOF‐801(Zr)/C/PVDF‐HFP MMM cell showed similar values between 1st cycle (4.326 Ω) and 50th cycle (4.248 Ω), evidencing its stability upon cycling. This can be attributed to the interlayer effectively limiting the diffusion of polysulfides. Figure 6e shows the rate performance of the cell with MOF‐801(Zr)/C/PVDF‐HFP MMM interlayer and Figure 6f the corresponding galvanostatic charge–discharge profiles at different current densities, where the enhancement of the cycling performance at low rates can be clearly seen when using the MOF‐based composite membrane. At higher rates, the improvement related to the blank cell is more limited, certainly due to the kinetics of the complex redox reaction of sulfur. Finally, when the current was switched back to 0.1 C, the cell could be recovered and maintained at 1013 mA h g^−1^ at the 30th cycle, indicating its excellent rate capability. The percentage retention is represented in Figure S16 (Supporting Information).

As mentioned above, the chain of the PVDF‐HFP polymer partially enters into the micropores of the MOF‐801(Zr), leading to a slight pore size narrowing. To evaluate if this would impact the Li^+^ diffusion within the membrane symmetric cells containing the MMM were assembled. The MOF‐801(Zr)/C/PVDF‐HFP MMM was paired between two stainless steel (SS) plates to make an SS | MMM | glass fiber (GF) | SS cell with electrolyte (1 M LiTFSI and 0.25 M LiNO_3_). The value of ionic conductivity can be calculated from EIS according to the following equation:

(1) σ=d/(RA)

*σ* is the ionic conductivity (S cm^−1^), *d* is the thickness of the separator (cm), *R* is the resistance (Ω), and *A* is the area of the stainless‐steel electrode (cm^2^).^[^ ^51^, ^52^ ^]^ As shown in Figure S17 (Supporting Information), the EIS of the GF separator illustrates a larger resistance (10.44 Ω), while the optimized MMM interlayer with the GF separator shows a resistance of 4.04 Ω. The calculated Li^+^ conductivity for this interlayer with the GF separator was 19.03 mS cm^−1^, which is higher than that of the GF separator (7.22 mS cm^−1^), indicating that the Li^+^ percolates faster when the MOF‐801(Zr)/C/PVDF‐HFP MMM interlayer was added than when only GF separator is used. Compared with the macropores in the original GF separator which allow solvated Li^+^ to pass through, the sub‐nanopores in the MOF/C‐based MMM are smaller than the diameters of solvated Li^+^. This is likely to cause the desolvation of Li^+^ when diffusing through the sub‐nanochannels under an electric field, which might facilitate subsequent fast Li^+^ conduction.^[^ ^46^, ^53^, ^54^ ^]^

**Figure 7:**
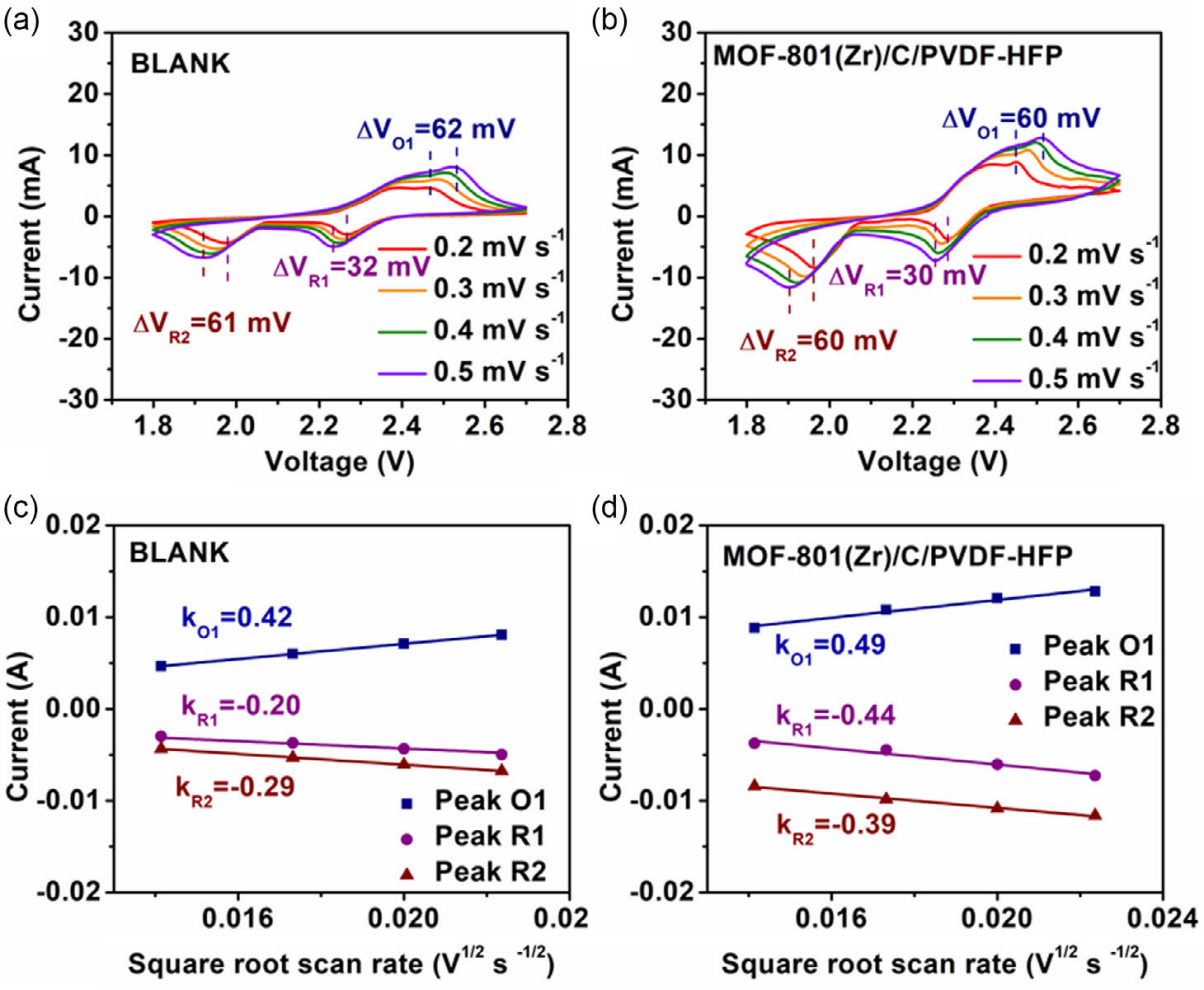
a) CV plots of Li–S batteries; b) CV plots of Li–S batteries with MOF‐801(Zr)/C/PVDF‐HFP MMM interlayer at different scan rates; c) corresponding linear fits of the peak current of CV plots of blank cell; and d) corresponding linear fits of the peak current of CV plots of cell with MOF‐801(Zr)/C/PVDF‐HFP MMM interlayer.

To further evaluate the charge‐transport characteristics, CV measurements under different scan rates from 0.2 to 0.5 mV s^−1^ (**Figure** **7**) were conducted. A distinguishable peak shift could be observed with the increase of the scan rate, leading to an increase of the polarization voltage at higher rates. It is clear that the cell with our hybrid interlayer showed a smaller peak shift with increasing scanning rate for O1 peaks (60 mV), R1 peaks (30 mV), and R2 peaks (60 mV) than without interlayer for O1 peaks (62 mV), R1 peaks (32 mV) and R2 peaks (61 mV), suggesting improved kinetics for Li^+^ transport and lithiation/delithiation process.^[^ ^55^, ^56^ ^]^ Lithium‐ion diffusion coefficient D_Li+_ (cm^2^ s^−1^) was calculated according to the Randles–Sevick equation:

(2) $$I_{p}=(2.687\times 10^{5})\;n^{3/2}{\text{A C}}_{\text{Li+}}{\left(D_{\text{Li+}}v\right)}^{1/2}$$

in which *I*_p_ (in A) is the peak current, *n* represents the number of electrons in the reaction (for Li–S batteries, *n* = 2), A (in cm^2^) indicates the electrode area (1.327 cm^2^), C_Li+_ (mol mL^−1^) means the lithium‐ion concentration, and v stands for the scanning rate (V s^−1^).^[^ ^57^, ^58^, ^59^ ^]^ As shown in **Table** **1**, the values of D_Li+_ for the cell with the MMM interlayer at all three peaks in CV curves show higher D_Li+_ when compared with those of blank cell, further demonstrating the faster Li^+^ diffusion rate and reaction kinetics. This may be due to the existence of abundant polar groups such as –OH on MOFs, which are beneficial to the reaction kinetics of Li^+^.^[^ ^11^ ^]^

**Table 1:** The Li^+^ diffusion coefficient.

Li^+^ diffusion coefficient [1 × 10^−8^ cm^2 ^s^−1^]	Blank	MOF‐801(Zr)/C/PVDF‐HFP MMM
Peak O1	11.10	15.11
Peak R1	2.52	12.18
Peak R2	5.29	9.57

## 3 Conclusion

We have designed a new type of freestanding microporous zirconium MOFs‐based MMM interlayer based on nanoparticles of the Zr fumarate MOF‐801, a KB porous carbon, and PVDF‐HFP. We could first demonstrate through a combination of experimental data from solid‐state NMR and MD calculations that the host–guest interaction between the polysulfides and the MOF structure relies not only in the polar nature of the acidic Zr‐OH terminal groups but also in the polar character of the carboxylate moieties of the fumarate linker. Incorporating MOF‐801(Zr) and KB into a PVDF‐HFP matrix led to a hybrid interlayer in Li–S batteries, with a remarkable improvement in the initial capacity up to 1110 mA h g^−1^, which was retained at a significant 880 mA h g^−1^ after 50 cycles. Electrochemical advanced characterizations demonstrated that the MOF‐based interlayer facilitates the diffusion of Li^+^ while effectively anchoring polysulfides and promoting their redox conversion, thus explaining the enhanced performance of the Li–S batteries. The development of MOF‐801(Zr)‐based MMM interlayers not only represents a promising approach for improving the performance of lithium–sulfur (Li–S) batteries, but these findings pave the way for a general strategy to prepare advanced MOF MMMs with enhanced electrochemical properties. By considering different MOF compositions and fillers, one could design a series of customized multifunctional interlayers with superior performance in Li–S batteries according to specific requirements in energy storage applications and beyond.

## 4 Experimental Section

4.1

4.1.1

##### Preparation of MOF‐801(Zr) Nanoparticles

Synthesis of nanoparticles of MOF‐801(Zr): 10.86 mmol of ZrOCl_2_·8H_2_O, 7.624 mmol of fumaric acid, 9 mL of formic acid, and 40 mL of deionized water were added in the reactor.^[^ ^32^ ^]^ The solution became cloudy within 5 h, indicating the formation of MOF‐801(Zr) nanoparticles. The product was collected by centrifugation, and washed with water and ethanol. The prepared MOF‐801 was finally dried at room temperature100 °C under the vacuum overnight before use.

##### Preparation of MMMs

MOF‐801(Zr), KB carbon, and acetone were added to a glass vial and then sonicated to obtain a uniform suspension. After that, 5% PVDF‐HFP in acetone solution was added to the MOF suspension. After stirring, the homogeneous MOF‐801(Zr)/C/PVDF‐HFP slurry was cast onto a glass substrate with a micrometric film applicator (Elcometer 3570/1). The coated membranes were dried in the air. See Table S1 (Supporting Information) for detailed information.

##### Preparation of Sulfur Cathodes and Assembly of Li–S Cells

C/S composites were prepared by mixing acetylene carbon black and sulfur powder in a mass ratio of 4:6. The mixture was heated to 160 °C at the rate of 1 °C min^−1^, then cooled to room temperature. As prepared C/S composites and PTFE (10%w PTFE) with a mass ratio of 0.95: 0.05 were mixed in EtOH. After grinding, the uniform pastry was rolled on a flat surface until it became homogeneous, then punched to 1.327 cm^2^, and finally vacuum dried at 80 °C overnight. The average sulfur loading for each cathode was ≈2.5 mg cm^−2^.

The Li–S cells were assembled in an argon‐filled glovebox. CR2032‐type coin cells were used, with S/C composite as the cathode, the MOF‐based interlayer directly placed after the cathode, the routine glass fiber separator, and finally lithium metal as the anode. The electrolyte was 1 M LiTFSI (lithium bis(trifluoromethanesulfonyl)imide) in 1,2‐dimethoxyethane (DME) and 1,3‐dioxolane (DOL) (1:1 v/v) with 0.25 M LiNO_3_ as an additive. The amount of electrolyte added to each cell was 15 μL electrolyte per 1 mg sulfur.

##### Material Characterization

All chemicals were purchased from commercial suppliers and used as received without further purification. PXRD data were recorded on a high‐throughput Bruker D8 Advance diffractometer working on transmission mode and equipped with a focusing Göbel mirror producing CuKα radiation (*λ* = 1.5418 Å) and a LynxEye detector. Nitrogen porosimetry data were collected on a Micromeritics Tristar instrument and Micromeritics Triflex adsorption analyzer at 77 K (preactivating at 373 K 12 h). SEM observations were performed using the FEI Magellan 400 scanning electron microscope. TEM images were recorded with a JEOL 2010 transmission electron microscope operating at 200 kV. TGA data were collected on Mettler Toledo TGA/DSC 2, STAR System apparatus with a 5 °C min^−1^ heating rate under oxygen flow. The self‐sustained films were cast using an Elcometer 3570/1 micrometric film applicator, which can be adjusted in 1 micron intervals, from 0 to 1 mm using a micrometric screw. A Büchi glass oven B‐585 was used for drying and vacuum transfer. Ultraviolet spectroscopy measurements were performed in quartz cells and in a UV‐1800 Shimadzu spectrometer.

##### Adsorption Tests of Li_2_S_6_

The 50 mM Li_2_S_6_ electrolyte was obtained by mixing 1 mmol of Li_2_S and 5 mmol of S. 10 mL of DOL, and 10 mL of DME were added to the vial and the solution was heated under stirring at 70 °C for 24 h in an argon‐filled glovebox. The 50 mg as‐prepared MOF samples were added into 1 mL of 0.05 M Li_2_S_6_ solution to confirm polysulfides adsorption.

##### Solid‐State NMR

The solid‐state NMR spectra were acquired at an 18.8 T/800 MHz spectrometer equipped with a Bruker Avance Neo console. The cross‐polarization (CP) contact time used in the ^1^H‐^13^C heteronuclear correlation (HETCOR) and ^7^Li‐^1^H CP‐heteronuclear single‐quantum coherence^[^ ^60^ ^]^ experiments are 2.5 ms and 200 μs, respectively. We have implemented frequency‐switched Lee–Goldburg (FSLG) homonuclear ^1^H decoupling during the ^1^H evolution period to achieve narrower lines in HETCOR.^[^ ^61^ ^]^ Note that the ^1^H chemical shift dimension is corrected by a numerical factor of $\cos\left(54.7^{\circ }\right)$ due to FSLG. The CODEX^[^ ^62^ ^]^ experiments were performed on a 3.2 mm HXY MAS probe. The ^7^Li‐^13^C rotational echo double resonance (REDOR) experiments were performed on a 2.5 mm HXY MAS probe. In the REDOR experiment,^[^ ^63^ ^]^ the ^1^H‐^13^C CP was first used to polarize ^13^C, followed by ^13^C‐^7^Li recoupling π pulses applied on ^7^Li channel with ^13^C detection. The MAS spinning frequencies are 10 kHz in all experiments. The ^1^H and ^13^C chemical shifts were referenced to the adamantine signal at 38.5 ppm.

##### MD Simulations

To probe the interactions between the MOF‐801 host material and Li_2_S_6_ guest molecules, first‐principles MD simulations were performed with the CP2K quantum chemistry software package.^[^ ^64^ ^]^ The molecular interactions were modeled with density functional theory (DFT) using the Perdew‐Burke‐Ernzerhof (PBE) functional^[^ ^65^ ^]^ with DFT‐D3(BJ) dispersion corrections.^[^ ^66^ ^]^ All the simulations were performed in the (N, P, $\sigma_{a}=0$, T) ensemble so that the shape of the unit cell is flexible. The temperature of the simulations was set to 300 K, while the pressure was set to 1 bar. The temperature and pressure were, respectively, controlled by a Nosé–Hoover thermostat^[^ ^67^, ^68^ ^]^ with a time constant of 0.1 ps and an MTTK barostat^[^ ^69^ ^]^ with a time constant of 1 ps. The plane wave basis set used a cutoff of 800 Ry and was combined with the TZVP‐MOLOPT basis set^[^ ^70^ ^]^ and Goedecker, Teter, and Hutter (GTH) pseudopotentials.^[^ ^71^ ^]^ Each MD simulation is at least 5 ps long.

##### 3D X‐Ray Microscopy

The composite membranes were scanned by nano‐CT (SkyScan 2214, Bruker, Belgium) equipped with an X‐ray tube using a W source (operated at 110 kV and 170 μA) and a charge coupled device (CCD) detector. Images of the MOF‐801(Zr)/C/PVDF‐HFP MMM were obtained employing no filter and with a voxel resolution of 1.0 μm. The capillaries were rotated 360° during data acquisition. Images were taken every 0.15°, i.e., 2401 frames, with an exposure time of 3700 ms. The geometrical settings were an object‐to‐source distance of 44.52 mm and an object‐to‐detector distance of 353.3 mm.

The reconstruction of the CT was done using the NRecon software (version 2.1.0.0, Bruker) based on the Feldkamp algorithm. CTAn software (version 1.18.8.0, Bruker) was used for the 3D analysis. A region of interest representative of the sample was selected and, the images were binarized. The objects of interest were represented in white color. The quantitative analyses of the sample were then performed through the 3D plug‐in analysis. CTVox software (Bruker, version 3.3.0) was used for volume rendering.

##### Electrochemical Measurements

The electrochemical behavior was investigated in CR2032‐type coin cells through galvanostatic charge‐discharge measurements on a BioLogic MPG2 potentiostat, within a potential window of 1.8–2.7 V (vs Li/Li^+^). Cyclic voltammetry tests were conducted within a potential range of 1.8–2.7 V (vs Li/Li^+^) at different scanning rates, from 0.1 to 0.5 mV s^−1^. EIS was performed using potentiostatic mode at open circuit potential. The measurements were done by applying a sinusoidal voltage with an amplitude of 5 mV and a frequency range from 100 kHz to 10 mHz.

## Conflict of Interest

The authors declare no conflict of interest.

## Supporting information

Supplementary Material

## Data Availability

The data that support the findings of this study are openly available in ChemRxiv at https://doi.org/10.26434/chemrxiv‐2023‐brbfn, reference number 0.

## References

[1] P. G. Bruce , S. A. Freunberger , L. J. Hardwick , J.-M. Tarascon , Nat. Mater. 2012, 11, 19. 10.1038/nmat3191 22169914

[2] A. Manthiram , S. H. Chung , C. Zu , Adv. Mater. 2015, 27, 1980. 25688969 10.1002/adma.201405115

[3] G. Zhou , H. Chen , Y. Cui , Nat. Energy 2022, 7, 312.

[4] L. Wang , W. Hua , X. Wan , Z. Feng , Z. Hu , H. Li , J. Niu , L. Wang , A. Wang , J. Liu , Adv. Mater. 2022, 34, 2110279. 10.1002/adma.202110279 35102639

[5] H. Chen , J. Liu , X. Zhou , H. Ji , S. Liu , M. Wang , T. Qian , C. Yan , Chem. Eng. J. 2021, 404, 126470.

[6] X. Liu , T. Qian , J. Liu , M. Wang , H. Chen , C. Yan , Energy Storage Mater. 2019, 17, 260.

[7] Y. Huang , L. Lin , C. Zhang , L. Liu , Y. Li , Z. Qiao , J. Lin , Q. Wei , L. Wang , Q. Xie , Adv. Sci. 2022, 9, 2106004. 10.1002/advs.202106004 PMC9036004 35233996

[8] Z. Wang , Y. Li , H. Ji , J. Zhou , T. Qian , C. Yan , Adv. Mater. 2022, 34, 2203699. 10.1002/adma.202203699 35816349

[9] L. P. Hou , Z. Li , N. Yao , C. X. Bi , B. Q. Li , X. Chen , X. Q. Zhang , Q. Zhang , Adv. Mater. 2022, 34, 2205284. 10.1002/adma.202205284 36085249

[10] J. Liu , T. Qian , N. Xu , M. Wang , J. Zhou , X. Shen , C. Yan , Energy Storage Mater. 2020, 24, 265.

[11] J. Han , S. Gao , R. Wang , K. Wang , M. Jiang , J. Yan , Q. Jin , K. Jiang , J. Mater. Chem. A 2020, 8, 6661.

[12] Y. Zang , F. Pei , J. Huang , Z. Fu , G. Xu , X. Fang , Adv. Energy Mater. 2018, 8, 1802052.

[13] S.-H. Chung , A. Manthiram , J. Phys. Chem. Lett. 2014, 5, 1978. 26273884 10.1021/jz5006913

[14] Y.-S. Su , A. Manthiram , Nat. Commun. 2012, 3, 1166. 23132016 10.1038/ncomms2163

[15] L. Chen , H. Yu , W. Li , M. Dirican , Y. Liu , X. Zhang , J. Mater. Chem. A 2020, 8, 10709.

[16] Y. Peng , S. Sanati , A. Morsali , H. García , Angew. Chem. 2023, 135, e202214707. 10.1002/anie.202214707 36468543

[17] X. Ma , M. Lepoitevin , C. Serre , Mater. Chem. Front. 2021, 5, 5573.

[18] Y. Shen , A. Tissot , C. Serre , Chem. Sci. 2022, 13, 13978. 36540831 10.1039/d2sc04314a PMC9728564

[19] Y. He , D. Li , L. Wu , X. Yin , X. Zhang , L. H. Patterson , J. Zhang , Adv. Funct. Mater. 2023, 33, 2212277.

[20] Y. Zheng , S. Zheng , H. Xue , H. Pang , J. Mater. Chem. A 2019, 7, 3469.

[21] A. E. Baumann , X. Han , M. M. Butala , V. S. Thoi , J. Am. Chem. Soc. 2019, 141, 17891. 31600066 10.1021/jacs.9b09538

[22] Y. Peng , X. Wei , Y. Wang , W. Li , S. Zhang , J. Jin , ACS Nano 2022, 16, 8329. 35549179 10.1021/acsnano.2c02520

[23] A. Knebel , J. Caro , Nat. Nanotechnol. 2022, 17, 911. 35995854 10.1038/s41565-022-01168-3

[24] X.-J. Hong , T.-X. Tan , Y.-K. Guo , X.-Y. Tang , J.-Y. Wang , W. Qin , Y.-P. Cai , Nanoscale 2018, 10, 2774. 29323375 10.1039/c7nr07118c

[25] J. Zhou , X. Yu , X. Fan , X. Wang , H. Li , Y. Zhang , W. Li , J. Zheng , B. Wang , X. Li , J. Mater. Chem. A 2015, 3, 8272.

[26] W. Liu , Y. Mi , Z. Weng , Y. Zhong , Z. Wu , H. Wang , Chem. Sci. 2017, 8, 4285. 28626566 10.1039/c7sc00668c PMC5468994

[27] Y. Li , S. Lin , D. Wang , T. Gao , J. Song , P. Zhou , Z. Xu , Z. Yang , N. Xiao , S. Guo , Adv. Mater. 2020, 32, 1906722. 10.1002/adma.201906722 31957092

[28] Y. Fan , Z. Niu , F. Zhang , R. Zhang , Y. Zhao , G. Lu , ACS Omega 2019, 4, 10328. 31460126 10.1021/acsomega.9b00884 PMC6648104

[29] P. S. Bárcia , D. Guimarães , P. A. Mendes , J. A. Silva , V. Guillerm , H. Chevreau , C. Serre , A. E. Rodrigues , Microporous Mesoporous Mater. 2011, 139, 67.

[30] S. Dai , C. Simms , I. Dovgaliuk , G. Patriarche , A. Tissot , T. N. Parac‐Vogt , C. Serre , Chem. Mater. 2021, 33, 7057.

[31] G. Wißmann , A. Schaate , S. Lilienthal , I. Bremer , A. M. Schneider , P. Behrens , Microporous Mesoporous Mater. 2012, 152, 64.

[32] S. Dai , F. Nouar , S. Zhang , A. Tissot , C. Serre , Angew. Chem. 2021, 133, 4328. 10.1002/anie.202014184 33179846

[33] H. Furukawa , F. Gandara , Y.-B. Zhang , J. Jiang , W. L. Queen , M. R. Hudson , O. M. Yaghi , J. Am. Chem. Soc. 2014, 136, 4369. 24588307 10.1021/ja500330a

[34] Y. Wang , Z. Deng , J. Huang , H. Li , Z. Li , X. Peng , Y. Tian , J. Lu , H. Tang , L. Chen , Energy Storage Mater. 2021, 36, 466.

[35] X.-J. Hong , C.-L. Song , Y. Yang , H.-C. Tan , G.-H. Li , Y.-P. Cai , H. Wang , ACS Nano 2019, 13, 1923. 30763073 10.1021/acsnano.8b08155

[36] J. Dechnik , J. Gascon , C. J. Doonan , C. Janiak , C. J. Sumby , Angew. Chem., Int. Ed. 2017, 56, 9292. 10.1002/anie.201701109 28378379

[37] G. Jin , J. Zhang , B. Dang , F. Wu , J. Li , Front. Chem. Sci. Eng. 2022, 16, 511.

[38] D. K. Sannes , S. Øien‐Ødegaard , E. Aunan , A. Nova , U. Olsbye , Chem. Mater. 2023, 35, 3793.

[39] M. U. Patel , R. Demir‐Cakan , M. Morcrette , J. M. Tarascon , M. Gaberscek , R. Dominko , ChemSusChem 2013, 6, 1177. 23749434 10.1002/cssc.201300142

[40] Q. Zou , Y.-C. Lu , J. Phys. Chem. Lett. 2016, 7, 1518. 27050386 10.1021/acs.jpclett.6b00228

[41] H. Wang , W. Zhang , J. Xu , Z. Guo , Adv. Funct. Mater. 2018, 28, 1707520.

[42] C. Amerein , U. Banerjee , Z. Pang , W. Lu , V. Pimenta , K. O. Tan , J. Magn. Reson. 2023, 348, 107391. 36801500 10.1016/j.jmr.2023.107391

[43] Z. Wan , D. Lei , W. Yang , C. Liu , K. Shi , X. Hao , L. Shen , W. Lv , B. Li , Q. H. Yang , Adv. Funct. Mater. 2019, 29, 1805301.

[44] P. Duan , J. C. Moreton , S. R. Tavares , R. Semino , G. Maurin , S. M. Cohen , K. Schmidt‐Rohr , J. Am. Chem. Soc. 2019, 141, 7589. 30973014 10.1021/jacs.9b02789

[45] R. Semino , J. C. Moreton , N. A. Ramsahye , S. M. Cohen , G. Maurin , Chem. Sci. 2018, 9, 315. 29629100 10.1039/c7sc04152g PMC5868319

[46] J. Chmiola , G. Yushin , Y. Gogotsi , C. Portet , P. Simon , P.-L. Taberna , Science 2006, 313, 1760. 16917025 10.1126/science.1132195

[47] W. Li , Y. Li , J. Caro , A. Huang , J. Membr. Sci. 2022, 643, 120021.

[48] H. Xu , F. Zhong , F. Chen , T.-X. Luan , P. Li , S. Xu , J. Gao , J. Mater. Chem. C 2022, 10, 7469.

[49] N. Widiastuti , T. Gunawan , H. Fansuri , W. N. W. Salleh , A. F. Ismail , N. Sazali , Membranes 2020, 10, 267. 32998417 10.3390/membranes10100267 PMC7599519

[50] W. G. Lim , C. Jo , A. Cho , J. Hwang , S. Kim , J. W. Han , J. Lee , Adv. Mater. 2019, 31, 1806547. 10.1002/adma.201806547 30484914

[51] W. Liu , K. Zhang , L. Ma , R. Ning , Z. Chen , J. Li , Y. Yan , T. Shang , Z. Lyu , Z. Li , Energy Storage Mater. 2022, 49, 1.

[52] Z. A. Ghazi , X. He , A. M. Khattak , N. A. Khan , B. Liang , A. Iqbal , J. Wang , H. Sin , L. Li , Z. Tang , Adv. Mater. 2017, 29, 1606817. 10.1002/adma.201606817 28318064

[53] H. Zhang , J. Hou , Y. Hu , P. Wang , R. Ou , L. Jiang , J. Z. Liu , B. D. Freeman , A. J. Hill , H. Wang , Sci. Adv. 2018, 4, eaaq0066. 29487910 10.1126/sciadv.aaq0066 PMC5817922

[54] Z. Chang , Y. Qiao , H. Deng , H. Yang , P. He , H. Zhou , Joule 2020, 4, 1776.

[55] T. Lei , Y. Xie , X. Wang , S. Miao , J. Xiong , C. Yan , Small 2017, 13, 1701013. 10.1002/smll.201701013 28748580

[56] T. Lei , W. Chen , W. Lv , J. Huang , J. Zhu , J. Chu , C. Yan , C. Wu , Y. Yan , W. He , Joule 2018, 2, 2091.

[57] N. H. Kwon , H. Yin , T. Vavrova , J. H. Lim , U. Steiner , B. Grobéty , K. M. Fromm , J. Power Sources 2017, 342, 231.

[58] T.-S. Chung , L. Y. Jiang , Y. Li , S. Kulprathipanja , Prog. Polym. Sci. 2007, 32, 483.

[59] W. Li , J. Qian , T. Zhao , Y. Ye , Y. Xing , Y. Huang , L. Wei , N. Zhang , N. Chen , L. Li , Adv. Sci. 2019, 6, 1802362. 10.1002/advs.201802362 PMC6702624 31453053

[60] E. Barbet‐Massin , A. J. Pell , J. S. Retel , L. B. Andreas , K. Jaudzems , W. T. Franks , A. J. Nieuwkoop , M. Hiller , V. Higman , P. Guerry , J. Am. Chem. Soc. 2014, 136, 12489. 25102442 10.1021/ja507382j PMC4156866

[61] G. P. Holland , T. M. Alam , J. Magn. Reson. 2006, 181, 316. 16798032 10.1016/j.jmr.2006.05.017

[62] E. R. D. Azevêdo , W. Hu , T. J. Bonagamba , K. Schmidt‐Rohr , J. Am. Chem. Soc. 1999, 121, 8411.

[63] T. Gullion , J. Schaefer , Advances in Magnetic and Optical Resonance, Vol. 13, Elsevier, https://www.google.com/search?sca_esv=7d380bcdbd6e9b9f&sca_upv=1&rlz=1C1GCEU_enIN1078IN1078&q=Amsterdam&si=AKbGX_obynita‐BNzxZrFs5Xp6xpKKQhp3e6ZAETk9w76AgBhATfr‐0uD9j0WKn96jQoxUYlD3lPmvUwLn2W_ZazhiO8YKrrlCUOPRG5MkaKYFwtaoDOHih64hiQ6tsQZ8RObK24Dn_XpIhkaim5c68CU4e1bMbdeHz_3v7HHod1DXvhBnlsaTGnAdrN0BWs0dxBbo7‐T11Q&sa=X&ved=2ahUKEwips7GJps2FAxUMwjgGHZ1ADwIQmxMoAXoECCYQAw, Amsterdam 1989.

[64] T. D. Kühne , M. Iannuzzi , M. Del Ben , V. V. Rybkin , P. Seewald , F. Stein , T. Laino , R. Z. Khaliullin , O. Schütt , F. Schiffmann , J. Chem. Phys. 2020, 152, 194103. 33687235 10.1063/5.0007045

[65] J. P. Perdew , K. Burke , M. Ernzerhof , Phys. Rev. Lett. 1996, 77, 3865. 10062328 10.1103/PhysRevLett.77.3865

[66] S. Grimme , S. Ehrlich , L. Goerigk , J. Comput. Chem. 2011, 32, 1456. 21370243 10.1002/jcc.21759

[67] S. Nosé , Mol. Phys. 1984, 52, 255.

[68] S. Nosé , J. Chem. Phys. 1984, 81, 511.

[69] G. J. Martyna , D. J. Tobias , M. L. Klein , J. Chem. Phys. 1994, 101, 4177.

[70] J. VandeVondele , J. Hutter , J. Chem. Phys. 2007, 127, 114105. 17887826 10.1063/1.2770708

[71] S. Goedecker , M. Teter , J. Hutter , Phys. Rev. B 1996, 54, 1703. 10.1103/physrevb.54.1703 9986014

