# Supplementary material for: Unraveling the Mechanisms of Zirconium Metal–Organic Frameworks‐Based Mixed‐Matrix Membranes Preventing Polysulfide Shuttling
Source: Small Sci. 2024 May 2;4(6):2300339. doi: 10.1002/smsc.202300339 (PMC11935271; doi:10.1002/smsc.202300339)
Supplement: Supplementary file 1 — Supplementary Material [file SMSC-4-2300339-s001.pdf]

Supporting Information

Unraveling the mechanisms of Zirconium MOFs based Mixed Matrix Membranes Preventing Polysulfide Shuttling

Wenqing Lu, Zhenfeng Pang, Aran Lamaire, Fu Liu, Shan Dai, Moisés L. Pinto, Rezan Demir-Cakan, Kong Ooi Tan, Veronique Van Speybroeck, Vanessa Pimenta, and Christian Serre**

Table S1. Preparation of Mixed Matrix Membranes (MMMs)

	MOF-801(Zr) (mg)	C (mg)	5 % PVDF-HFP (g)	Acetone (mL)
C/PVDF-HFP MMM	0	56.3	2.625	4.136
MOF-801(Zr)/PVDF-HFP MMM	56.3	0	2.625	4.136
MOF-801(Zr)/C/PVDF-HFP MMM	56.3	56.3	1.5	5.5

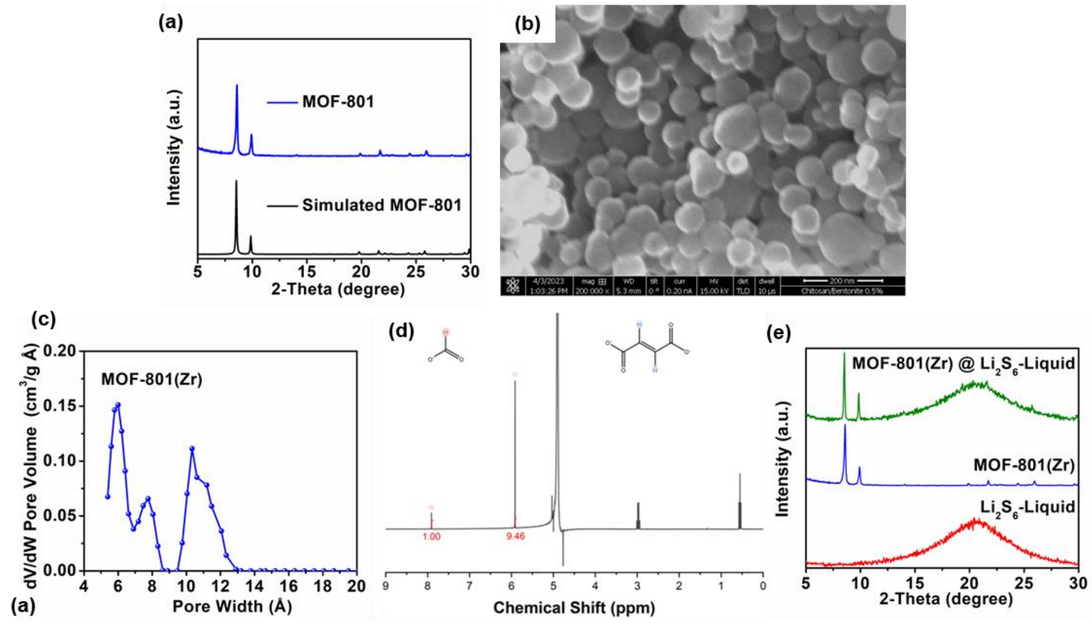

Figure S1. a) PXRD pattern of MOF-801(Zr) (λCu~1.5406 Å); b)) SEM image of MOF-801(Zr); c) Pore size distribution curve of the samples calculated from NLDFT-N2 method); d) ^{1}H NMR spectra of the digested MOF-801 (by using KOH) in D_2O; e) XRD pattern of wet samples.

	**PVDF-HFP membrane**	**MOF-801(Zr)/PVDF-HFP MMM**
**Beginning**	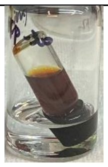	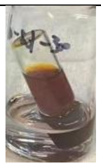
**30 min**	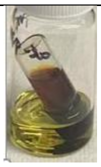	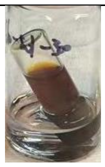
**1 h**	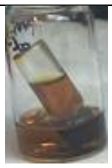	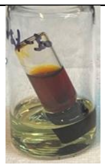

Figure S2. Permeability test. The small vial covered with MOF-based composite membrane or PVDF-HFP membrane contains the 50 mM Li_2S_6 electrolyte, while the larger bottle contains 1,2-dimethoxyethane and 1,3-dioxolane (1:1 v/v) solution.

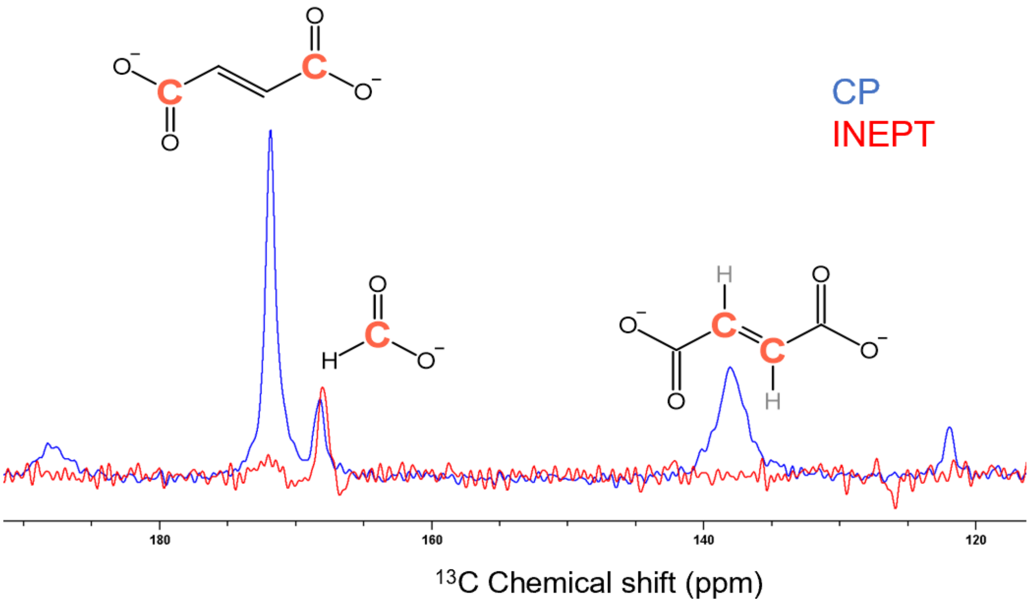

Figure S3. ^{1}H-^{13}C cross polarization and INEPT spectra. Only a single peak around 168 ppm appeared when the INEPT delay time was set to the value that corresponds to the ^{1}H-^{13}C *J* coupling strength (205 Hz) in formate.

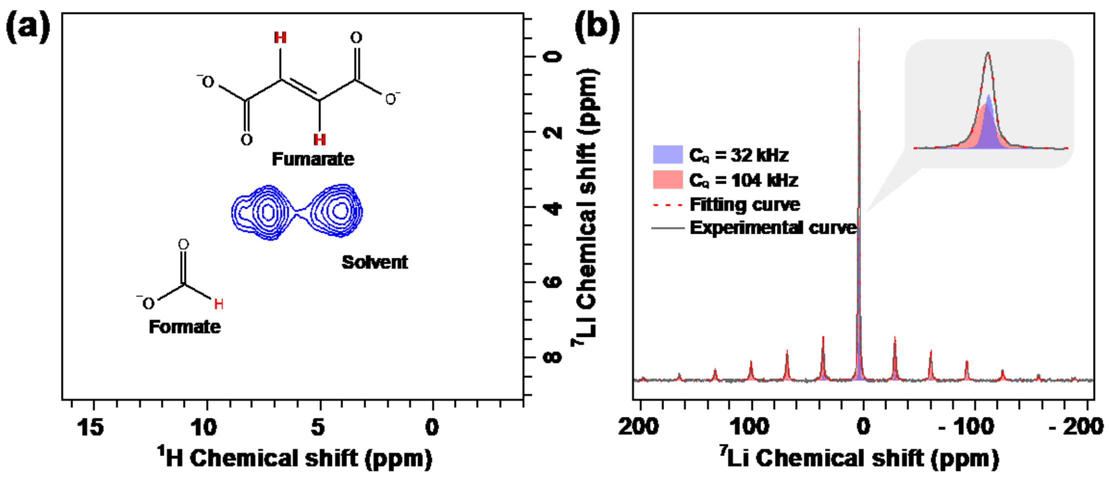

Figure S4. ^{7}Li spectra of Li_2S_6-loaded MOF-801. a) ^{1}H-^{7}Li HSQC spectrum, where we observed the cross peaks between lithium and all ^{1}H sites including formate, fumarate,

and solvent; b) ^{7}Li spin echo spectra and fitting results. A flexible Li site (C_Q = 32 kHz) and a more rigid Li site (C_Q = 104 kHz) with a ratio of 1:3 was used to fit the experimental data.

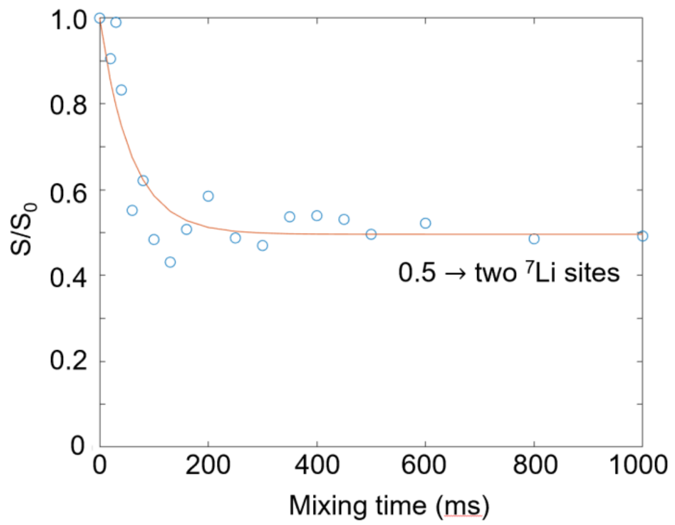

Figure S5. ^{7}Li CODEX curve. With long mixing time, the y-axis intensity, S/S_0, is around 0.5 which means there are two ^{7}Li close to each other.

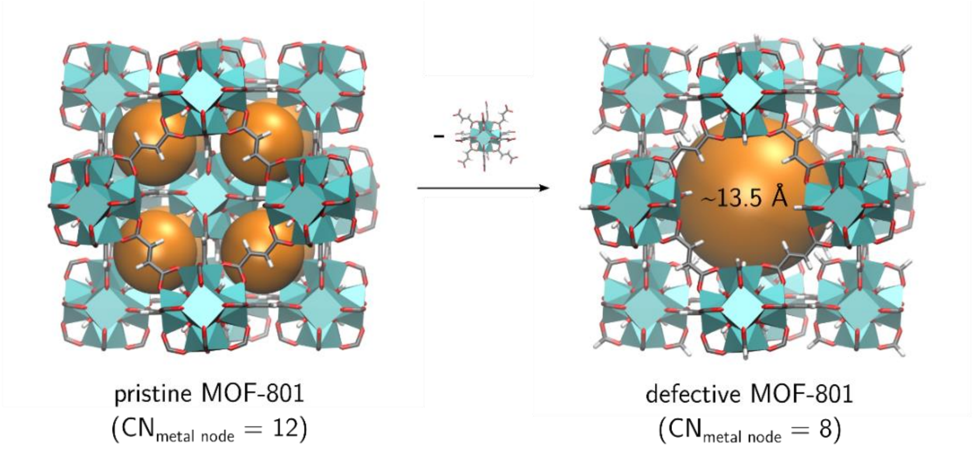

Figure S6. Visualization of the defective MOF-801 structure (with an 8-fold linker connectivity for every Zr_6 oxocluster) used in the MD simulations. The orange spheres represent the pores in the MOF-801 structure.

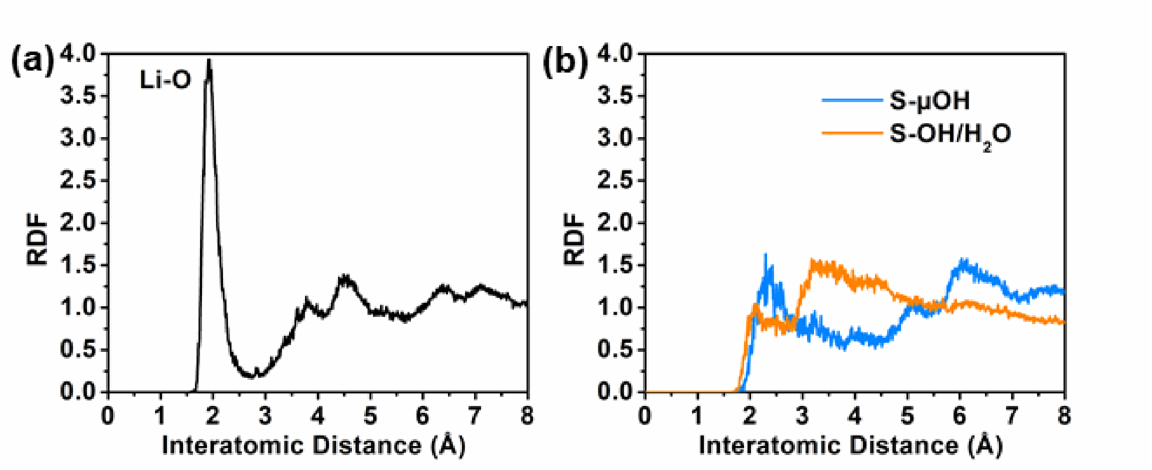

Figure S7. Radial distribution functions (RDFs) for a) Li-O and b) S-$H_{\mu OH}$ and S-$H_{OH/H2O}$ atom pairs obtained from first-principles MD simulations of MOF-801 (with a 9-fold linker coordination) containing 9 Li_2S_6 molecules.

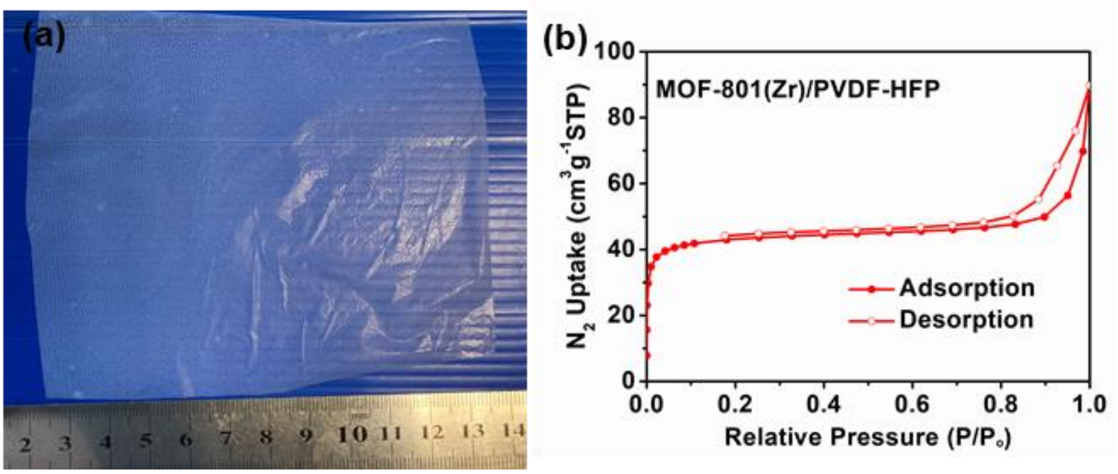

Figure S8. a) Photo of the 30% MOF-801(Zr)/70%PVDF-HFP MMMs; b) N_2 adsorption-desorption isotherms of 30%MOF-801/70%PVDF-HFP MMMs.

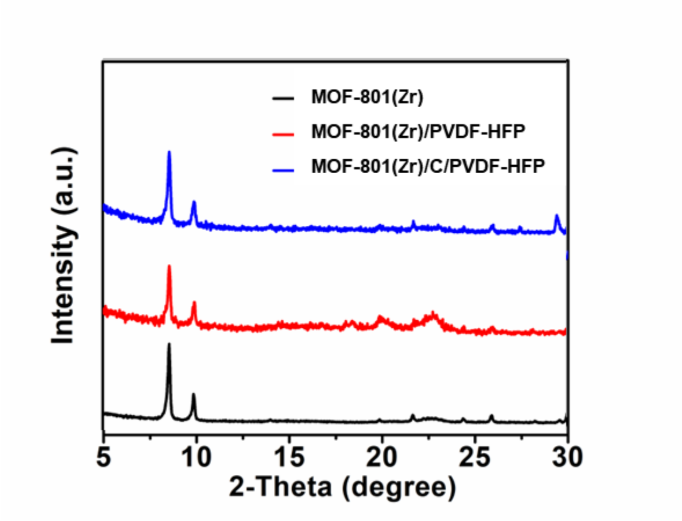

Figure S9. XRD pattern of different samples

Table S2. Preparation of MMMs

	MOF-801(Zr) (mg)	C (mg)	5 %PVDF-HFP solution(g)	Aceton (mL)
50% MOF-801(Zr) / 10% C /PVDF-HFP MMM	93.8	18.8	1.5	5.5
40% MOF-801(Zr) / 20% C /PVDF-HFP MMM	75.1	37.5	1.5	5.5
30% MOF-801(Zr) / 30% C /PVDF-HFP MMM	56.3	56.3	1.5	5.5

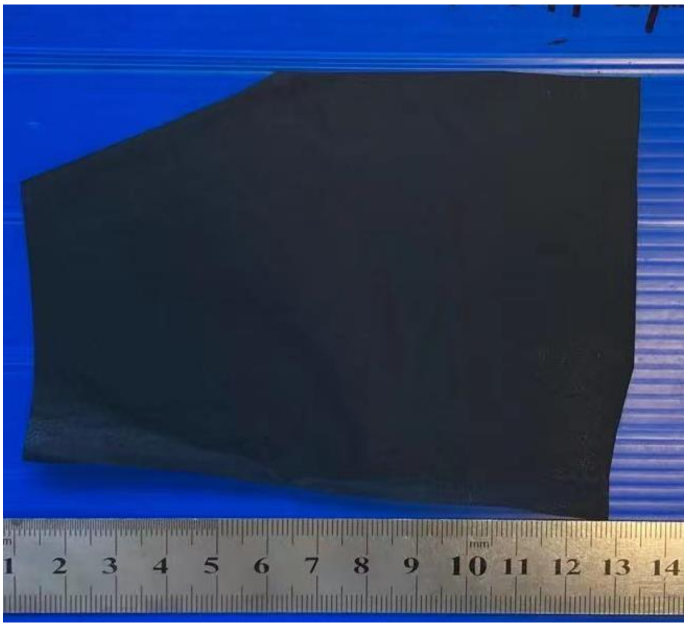

Figure S10. The 50% MOF-801(Zr) / 10% C / 40% PVDF-HFP MMM.

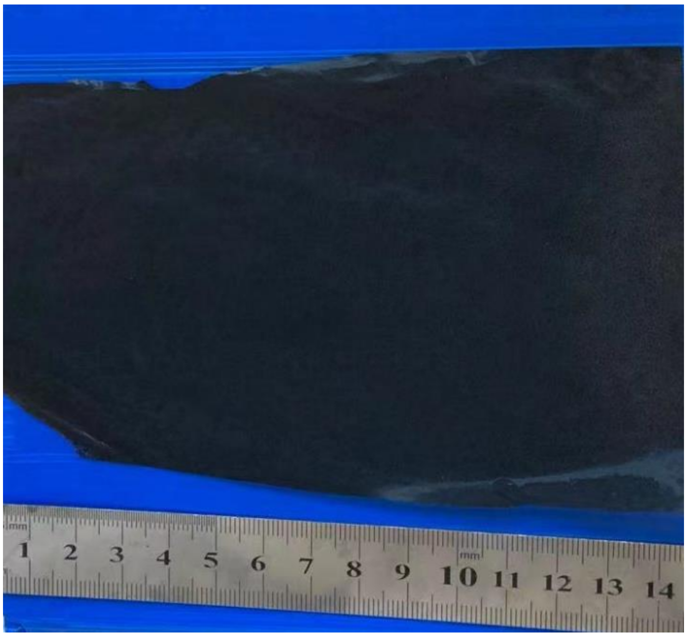

Figure S11. The 40% MOF-801 / 20% C / 40% PVDF-HFP MMM.

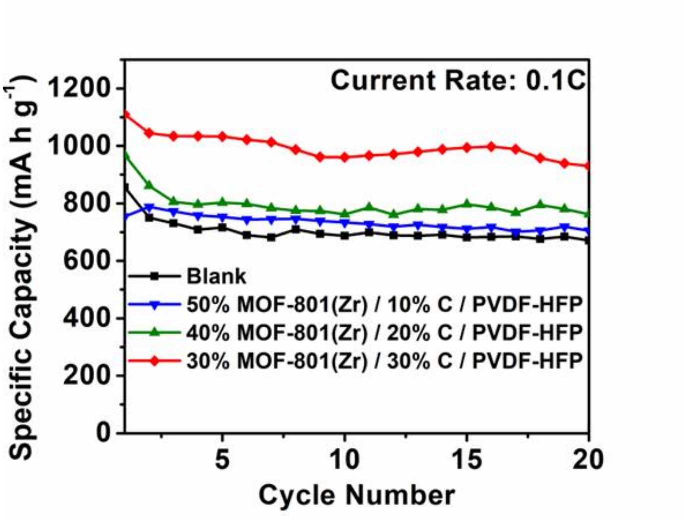

Figure S12. Galvanostatic cycling performance of Li-S cells with different carbon MOF contents MMMs at 0.1 C

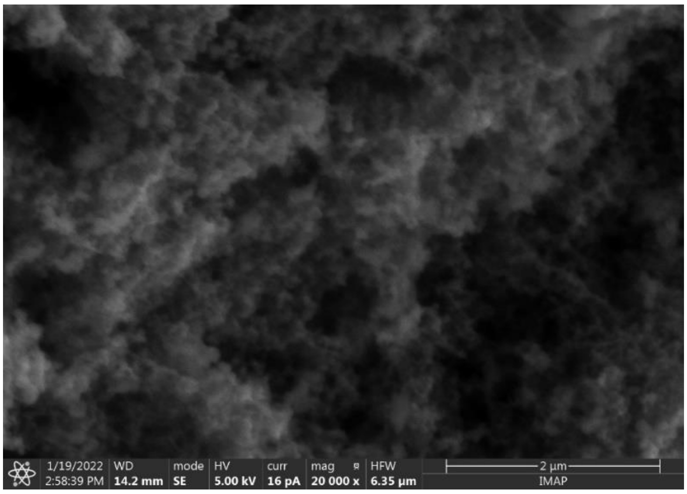

Figure S13. SEM image of the interior of the MOF-801(Zr)/C/PVDF-HFP MMM.

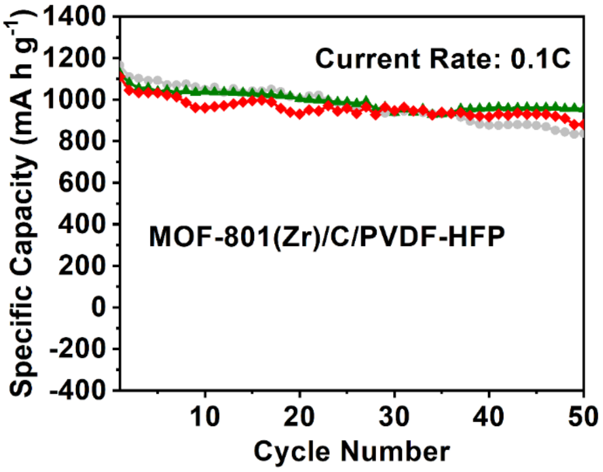

Figure S14. Galvanostatic cycling performance of three equivalent cells at 0.1 C.

Table S3. Fitting results achieved from EIS.

	R1	R2	R3
PVDF-HFP membrane	7.034	18.07	61.33
C/PVDF-HFP MMM	6.39	11.01	41.47
MOF-801(Zr)/PVDF-HFP MMM	4.923	6.863	30.06
MOF-801(Zr)/C/PVDF-HFP MMM	6.268	6.827	16.57

Table S4. Fitting results achieved from EIS.

MOF-801(Zr)/C/PVDF-HFP MMM	R1	R2	R3
Beginning	6.268	6.827	16.57
After 1st cycle	7.77	4.326	4.328
After 50th cycle	6.084	4.248	2.787

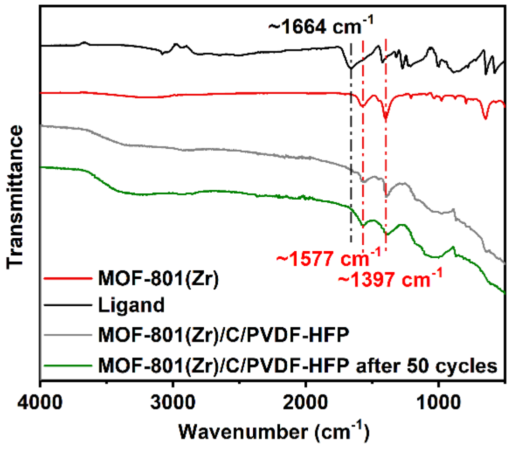

Figure S15. FT-IR spectra of MOF-801(Zr), Ligand, MOF-801(Zr)/C/PVDF-HFP MMMs, and MOF-801(Zr)/C/PVDF-HFP MMMs recovered after 50 cycles of the cell.

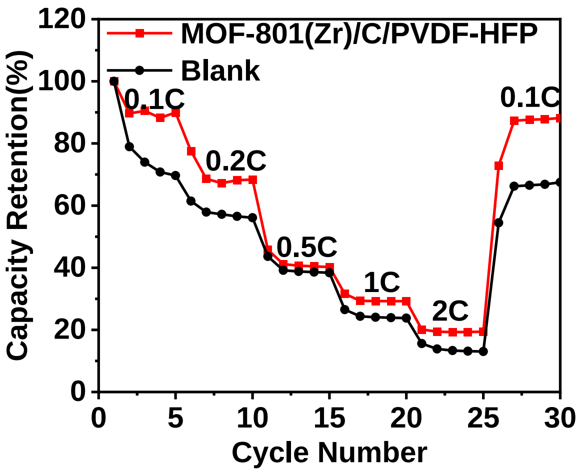

Figure S16. Capacity retention at different current densities.

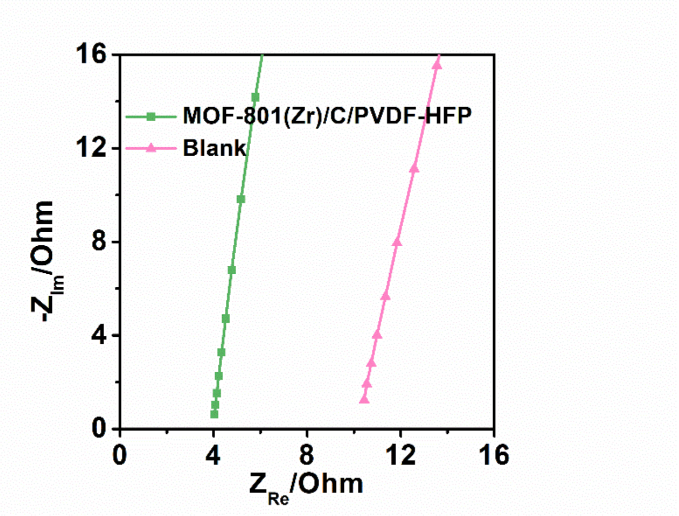

Figure S17. EIS plots of SS-SS symmetrical batteries with (green) and without (pink) MOF-801(Zr)/C/PVDF-HFP MMM interlayer.
